# NAMPT encapsulated by extracellular vesicles from young adipose-derived mesenchymal stem cells treated tendinopathy in a “One-Stone-Two-Birds” manner

**DOI:** 10.1186/s12951-022-01763-5

**Published:** 2023-01-05

**Authors:** Guanghao Wu, Qihang Su, Jie Li, Chao Xue, Jie Zhu, Qiuchen Cai, Jingbiao Huang, Shaoyang Ji, Biao Cheng, Hengan Ge

**Affiliations:** 1 School of Materials Science and Engineering, Beijing Institute of Technology, Beijing, 100081 China; 2 Department of Orthopedics, Shanghai Tenth People’s Hospital, School of Medicine, Tongji University, Shanghai, 200072 China; 3 Department of Orthopedics, Zhabei Central Hospital of Jing’an District, Shanghai, 200070 China; 4 National Key Laboratory of Biochemical Engineering, Institute of Process Engineering, Chinese Academy of Sciences, Beijing, 100190 China; 5 Department of Orthopedics, Tongji Hospital, School of Medicine, Tongji University, Shanghai, 200065 China

**Keywords:** Extracellular vesicles, Adipose tissue-derived mesenchymal stem cells, NAD+ metabolism, Macrophage, Tendinopathy

## Abstract

**Background:**

Tendinopathy is the leading sports-related injury and will cause severe weakness and tenderness. Effective therapy for tendinopathy remains limited, and extracellular vesicles (EVs) derived from adipose tissue-derived mesenchymal stem cells (ADMSCs) have demonstrated great potential in tendinopathy treatment; however, the influence of aging status on EV treatment has not been previously described.

**Results:**

In this study, it was found that ADMSCs derived from old mice (ADMSC^old^) demonstrated remarkable cellular senescence and impaired NAD+ metabolism compared with ADMSCs derived from young mice (ADMSC^young^). Lower NAMPT contents were detected in both ADMSC^old^ and its secreted EVs (ADMSC^old^-EVs). Advanced animal experiments demonstrated that ADMSC^young^-EVs, but not ADMSC^old^-EVs, alleviated the pathological structural, functional and biomechanical properties in tendinopathy mice. Mechanistic analyses demonstrated that ADMSC^young^-EVs improved cell viability and relieved cellular senescence of tenocytes through the NAMPT/SIRT1/PPARγ/PGC-1α pathway. ADMSC^young^-EVs, but not ADMSC^old^-EVs, promoted phagocytosis and M2 polarization in macrophages through the NAMPT/SIRT1/Nf-κb p65/NLRP3 pathway. The macrophage/tenocyte crosstalk in tendinopathy was influenced by ADMSC^young^-EV treatment and thus it demonstrated "One-Stone-Two-Birds" effects in tendinopathy treatment.

**Conclusions:**

This study demonstrates an effective novel therapy for tendinopathy and uncovers the influence of donor age on curative effects by clarifying the detailed biological mechanism.

**Graphical Abstract:**

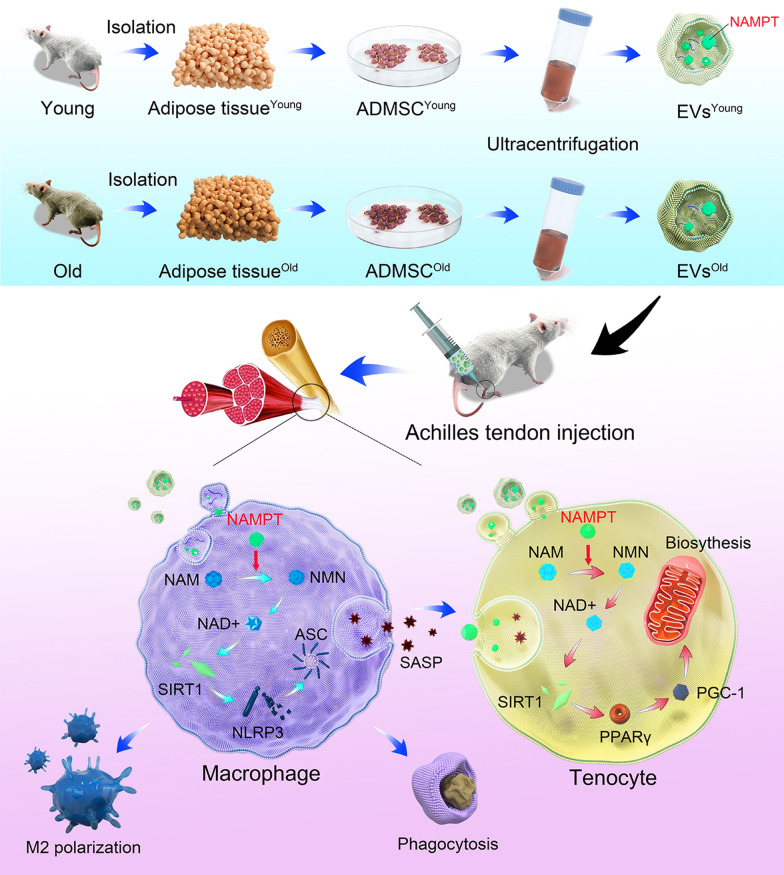

**Supplementary Information:**

The online version contains supplementary material available at 10.1186/s12951-022-01763-5.

## Introduction

Chronic tendinopathy, characterized by activity-related tendon pain with varying degrees of weakness, tenderness, and localized swelling, accounts for 30 ~ 50% of all sports-related injuries. Repeated pain and long-term dysfunctions affect approximately 30 million tendinopathy cases and lead to serious health loss and economic burden; however, there is currently no specific therapy [1]. Increased age and obesity cause tendon overuse, promote pathological degeneration and subsequently tendon rupture [2]. Conservative treatments for tendinopathy have relatively low healing ability because of the limited regeneration capacity of tendons. Arthroscopic surgery is a last resort for tendinopathy; however, a recent meta-analysis reported that surgery failed to provide more benefits over other therapeutic interventions, including eccentric exercises and injectables [3]. Considering the urgent requirements of novel treatments for tendinopathy, therapeutic options for both alleviating tendon pathological features and promoting recovery after surgery are needed.

Multiple threptic strategies, including corticosteroid injection and platelet-rich plasma application, have been recommended for managing tendinopathy [1, 4]. Stem cells, both tendon stem/progenitor cells (TSPCs) and mesenchymal stem cells (MSCs) from other tissues, have been extensively studied in tendon healing and tendinopathy treatments [5, 6]. Common sources of MSCs include bone marrow, fat, gums, and dental pulp; thus, it is natural that biological diversities exist among EVs derived from different MSCs [7]. Adipose tissue-derived mesenchymal stem cells (ADMSCs) demonstrate tissue repair potentials through differentiate into target cells in the injured area, secretion of various cytokines/growth factors to neighboring cells as well as immunomodulatory effects to reduce the inflammatory response in damaged tissues. Comparing with other MSCs, ADMSCs are relatively easy to obtain and demonstrate a higher yield and stable quality, thus are more commonly used to treat various disorders, including tendinopathy [8, 9]. Even MSCs are regarded as immunogenic and provide differentiation potential, considerable heterogeneity and a limited amount of cells infiltrating to target organs in MSC allogeneic transplantation [10]. Extracellular vesicles (EVs), which carry multiple types of bioactive factors, are nanosized lipid particles released by almost all cell types [11], and the EVs have been used as a novel cell-free therapy in the management of various disorders, including carcinoma, neurological and cardiovascular diseases and tendinopathy [12, 13].

EVs from MSCs educated by inflammation, hypoxia or mechanical stimuli provide highly modified secretomes and thus promote improved therapeutic efficacy [14, 15]. Both the characteristics and functions of EVs are also influenced by donors health issues, especially diabetes, obesity, cardiovascular disorders and aging [16, 17], and this is an important consideration for applying EVs as biomarkers or potential drugs. The expectation of a demographic shift to a higher elderly population structure in developed countries highlights the importance of senescence-related researches. Aging significantly influences the amount and biological function of EVs based on both clinical observations and experimental assays [18]. It has been reported that the beneficial effects of osteocyte-derived EVs diminished with aging [19], and similar outcomes were detected in MSCs [20]. A recent elegant showed that small extracellular vesicles (sEVs) derived from ADSCs of young animals significantly promoted health span through metabolome modification [21]. Mounting evidences demonstrated that aging status would influence the therapeutic effects, however, the impact of senescence in applying EVs, which is regarded as a potential therapy for tendinopathy, to treat tendinopathy remains unclear.

**Fig. 1:**
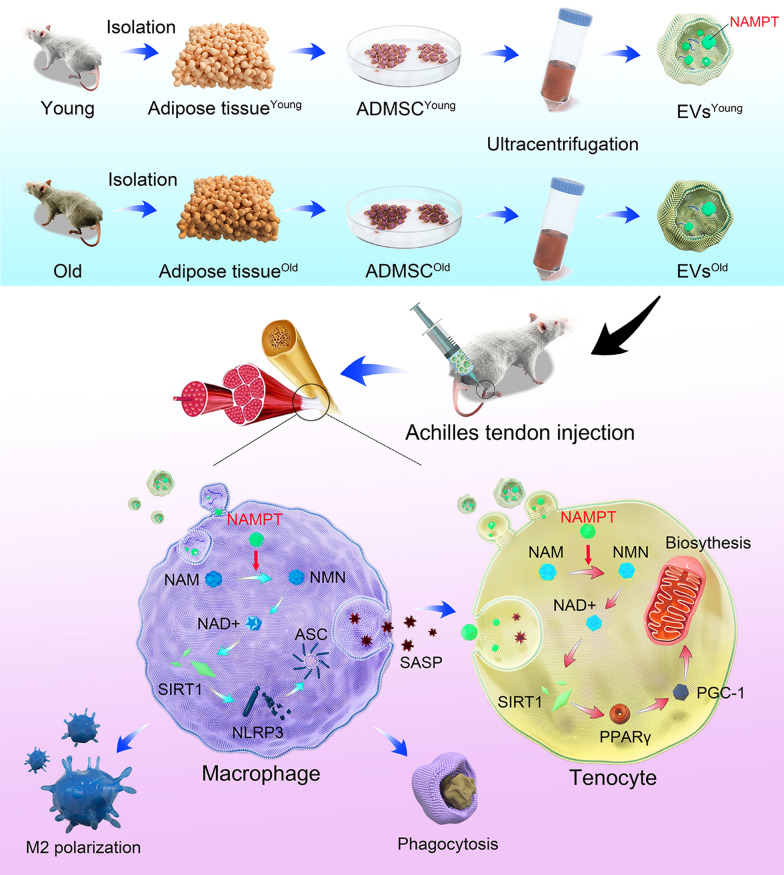
The illustration of "One-Stone-Two-Birds" strategy

Nicotinamide adenine dinucleotide (NAD+) is the core cofactor for mitochondrial function, and its progressive decline is closely related to the occurrence of degenerative diseases [22]. NAD+ metabolism is in dynamic equilibrium based on the balance of synthesis and degradation. The salvage synthesis pathway produces NAD+, accounting for 85% of the total human NAD+, and the NAD+ synthesis enzyme nicotinamide phosphoribosyl transferase (NAMPT) is the restriction enzyme of this cycle. Previous elegant studies have shown that extracellular NAMPT exists in EVs exclusively in both mice and humans, and a significantly decreased extracellular NAMPT content is detected in aged mice [23]. In this study, we comprehensively analyzed NAD+ metabolism-related enzymes in ADMSCs derived from young and old mice and then detected the NAMPT contents in EVs from different donors (Fig. 1). By detecting the effects of EVs derived from young or old mouse ADMSCs on tendinopathy, the concepts of EV therapy in tendinopathy management were updated. Both tenocytes and macrophages in tendinopathy status were protected by EVs derived from ADMSCs of young mice, and thus, we provided a potential "One-Stone-Two-Birds" strategy for tendinopathy treatment.

## Results

### ADMSC^old^ demonstrated significant cellular senescence and impaired NAD+ metabolism

**Fig. 2:**
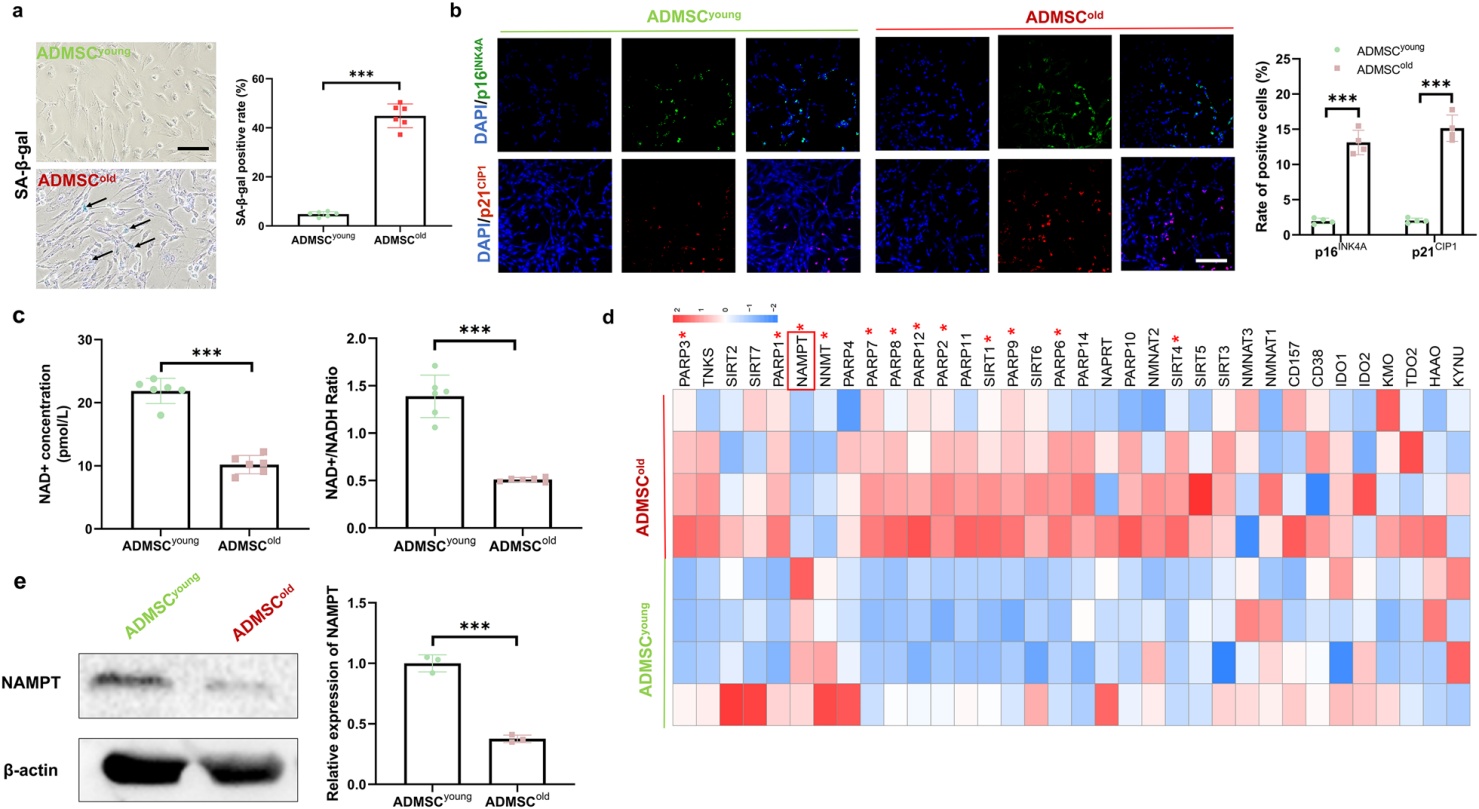
ADMSC^old^ demonstrated significant cellular senescence and impaired NAD+ metabolism. **a** SA-β-gal activity and the percentages of SA-β-gal-positive cells in ADMSC^young^ and ADMSC^old^. Scale bar = 200 μm, n = 6. **b** Immunofluorescence staining of p16^INK4A^ (green), p21^CIP1^ (red) and nuclear (DAPI, blue) in cultured ADMSC^young^ and ADMSC^old^. Scale bar = 200 μm. Quantification of the relative rate of positive cells was performed, n = 6. **c** NAD+ concentrations and NAD+/NADH ratios in ADMSC^young^ and ADMSC^old^, n = 6. **d** The relative expression of NAD+ metabolism-related genes by RT‒PCR in ADMSC^young^ and ADMSC^old^ and their expression patterns were visualized by a heatmap. Low expression is marked in blue, and high expression is marked in red. Red * indicates significantly different genes, n = 3. The red box highlights the target gene NAMPT, which was used in an advanced study. (e) Western blot analysis and quantification of the expression of NAMPT proteins in ADMSC^young^ and ADMSC^old^, n = 3. Data are presented as the mean ± SD (***: P < 0.001)

In this study, ADMSCs from both young mice (2 months old) and old mice (22 months old) were isolated and used in advanced assays. Because the donor mice were in different age stages, it is natural to hypothesize that ADMSC^old^ demonstrated a significant senescent status. Flow cytometric analysis revealed that ADMSC^young^ and ADMSC^old^ highly expressed ADMSCs markers CD29 (98.23% and 93.43%), CD90 (92.08% and 92.64%) but not negative factors CD45 (0.26% and 4.27%, Additional file 1: Figure S1), respectively. Through SA-β-gal staining (Fig. 2a) and immunofluorescence of cellular senescence-related markers, including p16^INK4A^ and p21^CIP1^ (Fig. 2b), it was found that a significantly evaluated senescence was detected in the ADMSC^old^ but not in the ADMSC^young^ (P < 0.001). Because NAD+ metabolism dysfunction was significantly related to cellular senescence, NAD+ contents were analyzed. Compared with the ADMSC^young^, a significantly decreased NAD+ content and NAD+/NADH content were detected in the ADMSC^old^ (P < 0.001, Fig. 2c). The basis of the physiological function of NAD+ was the dynamic equilibrium of biosynthesis and consumption; thus, the key genes related to NAD+ biogenesis and consumption were detected. As shown in Fig. 2d, NAD+ biosynthesis enzymes, including NAMPT and NNMT, were significantly downregulated in ADMSC^old^. In addition, NAD+ consumption-related genes, including PARP3, PARP1, PARP7, PARP8, PARP12, PARP2, PARP9, SIRT1 and SIRT4, were significantly upregulated (P < 0.05). Because NAMPT is the rate-limiting enzyme of the salvage pathway, it was chosen in the following study, and it was found that the NAPMT protein level was decreased (Fig. 2e, P < 0.001).

### NAMPT levels were decreased in isolated ADMSC^old^-EVs compared with ADMSC^young^-EVs

**Fig. 3:**
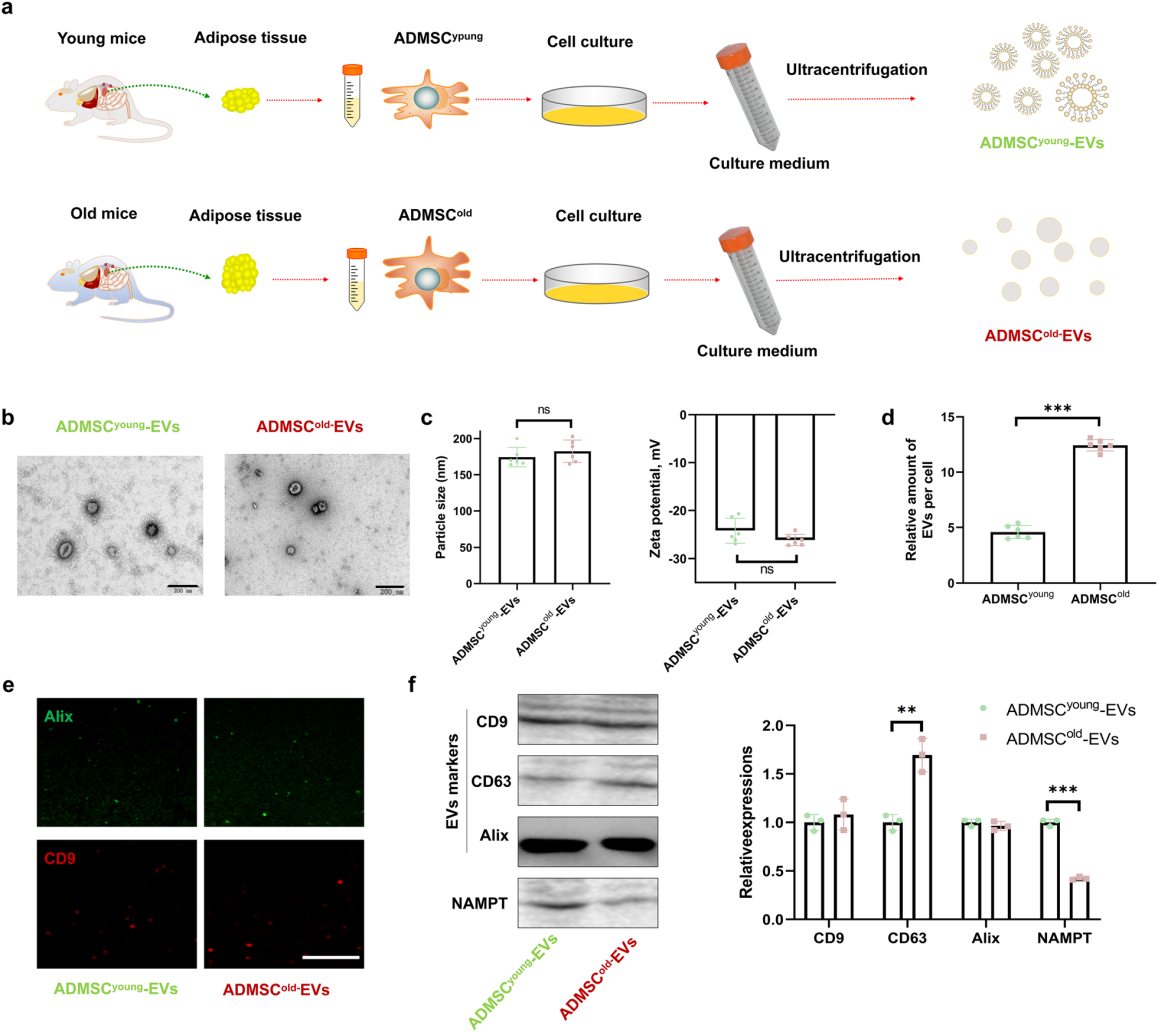
The NAMPT level was lower in isolated ADMSC^old^-EVs than in ADMSC^young^-EVs. **a** Schematic diagram of the isolation of ADMSC^young^-EVs and ADMSC^old^-EVs. **b** Morphological analysis of ADMSC^young^-EVs and ADMSC^old^-EVs by TEM. Scale bar = 200 nm. **c** Size distribution and zeta potential of ADMSC^young^-EVs and ADMSC^old^-EVs, n = 6. **d** Relative amount of secreted EVs in ADMSC^young^ and ADMSC^old^, n = 6. **e** Immunofluorescence staining of Alix (green) and CD9 (red) in ADMSC^young^-EVs and ADMSC^old^-EVs. Scale bar = 50 μm. **f** Western blot analysis and quantification of the expression of CD9, CD63, and Alix proteins as well as NAMPT in ADMSC^young^-EVs and ADMSC^old^-EVs, n = 3. Data are presented as the mean ± SD (ns: no significance; **: P < 0.01; ***: P < 0.001)

ADMSC^young^ and ADMSC^old^ were isolated for advanced EV preparations, and the isolated EVs were ADMSC^young^-EVs and ADMSC^old^-EVs (Fig. 3a). Biological diversity was the basis of the potential differential biological effects, and characterization of both types of EVs was the very first step. As observed under TEM, both ADMSC^young^-EVs and ADMSC^old^-EVs exhibited a cup-shaped morphology (Fig. 3b). Advanced DLS measurement revealed that neither the particle size (174.3 ± 13.47 vs. 182.5 ± 15.44 nm, P = 0.352) nor the zeta potential (− 24.20 ± 2.583 vs. − 26.12 ± 1.177 mV, P = 0.129) in ADMSC^young^-EVs and ADMSC^old^-EVs demonstrated a significant difference (Fig. 3c). The EV productivity of EVs in both ADMSCs was analyzed, and the obtained results demonstrated that ADMSC^old^ was more productive in EV generation (P < 0.001, Fig. 3d). For EV identification, immunofluorescence or immunoblotting of exosome markers, including CD9, Alix and CD63, was conducted (Fig. 3e, f). No significant difference was detected in EV marker expression between ADMSC^young^-EVs and ADMSC^old^-EVs (P > 0.05). Because adipose-derived EVs were reported to be the vector of extracellular NAMPT (eNAMPT) [23], the NAMPT contents in ADMSC^young^-EVs and ADMSC^old^-EVs were detected. As shown in Fig. 3f, a significantly decreased NAMPT content was detected in the ADMSC^old^-EVs, which demonstrated that only ADMSC^young^-EVs could provide a NAMPT regeneration effect.

### ADMSC^young^-EVs, but not ADMSC^old−^EVs, alleviated the pathological structural, functional and biomechanical properties in tendinopathy

**Fig. 4:**
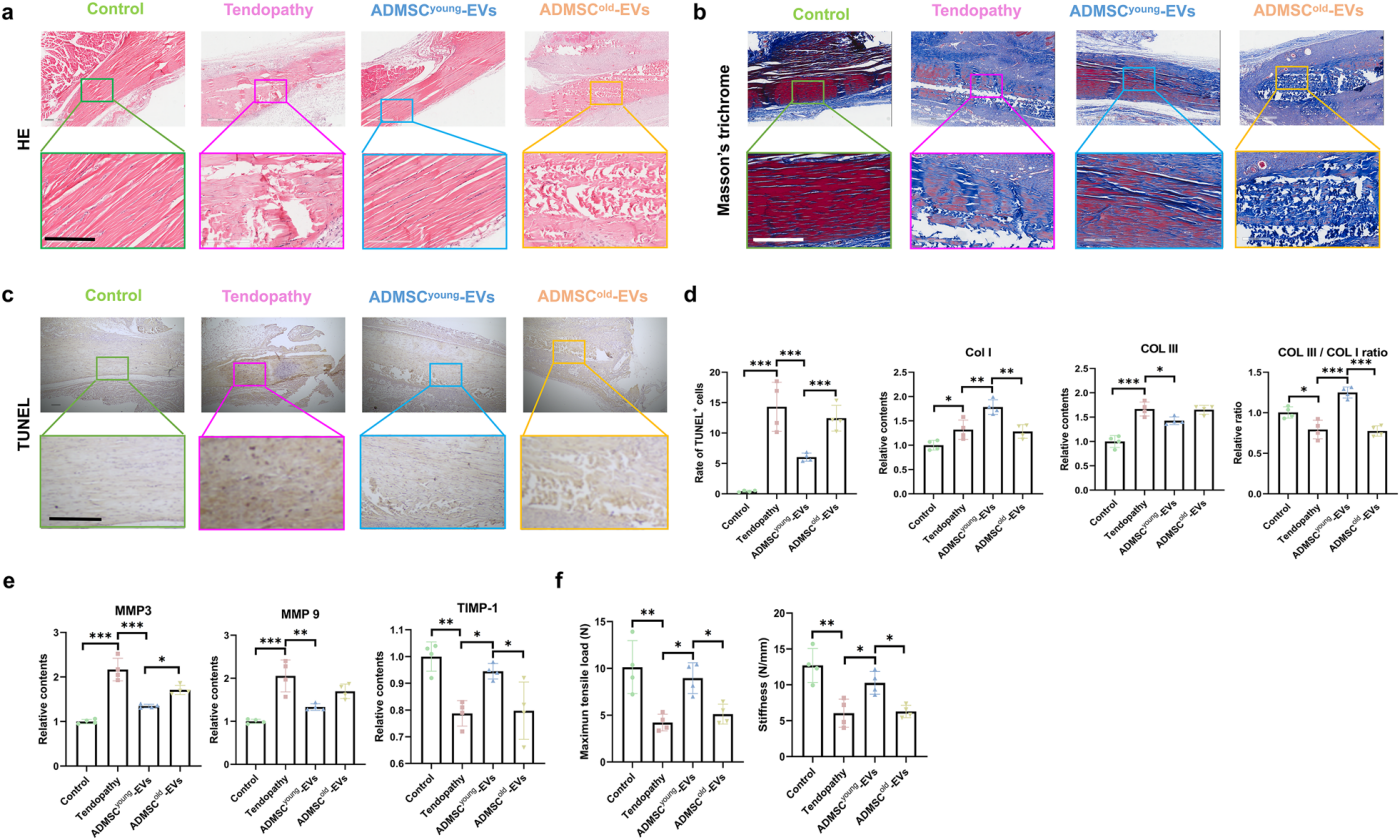
ADMSC^young^-EVs, but not ADMSC^old^-EVs, alleviated the pathological structural, functional and biomechanical properties in tendinopathy. **a** H&E staining of tendon tissues in the control, tendinopathy, tendinopathy with ADMSC^young^-EVs and tendinopathy with ADMSC^old^-EVs treatment groups 4 weeks after surgery. Scale bar = 200 μm, n = 4. **b** Masson’s trichrome staining of tendon tissues of the control, tendinopathy, tendinopathy with ADMSC^young^-EVs and tendinopathy with ADMSC^old^-EVs treatment groups 4 weeks after surgery. The collagen fibers were stained red, and the collagen matrix disruptions were stained blue. Scale bar = 200 μm, n = 4. **c** TUNEL assay of the control, tendinopathy, tendinopathy with ADMSC^young^-EVs and tendinopathy with ADMSC^old^-EVs treatment group 4 weeks after surgery. Scale bar = 200 μm. The rate of TUNEL-positive cells was analyzed, n = 4. **d** The relative expression of Col I and Col III and the Col III/Col I ratio by ELISAs of tendon tissues of the control, tendinopathy, tendinopathy with ADMSC^young^-EVs and tendinopathy with ADMSC^old^-EVs treatment groups 4 weeks after surgery, n = 4. **e** The relative expression of MMP3, MMP9 and TIMP-1 by ELISAs of tendon tissues of the control, tendinopathy, tendinopathy with ADMSC^young^-EVs and tendinopathy with ADMSC^old^-EVs treatment groups 4 weeks after surgery. **f** The biomechanical properties, including the maximum tensile load and stiffness of tendon tissues of the control, tendinopathy, tendinopathy with ADMSC^young^-EVs and tendinopathy with ADMSC^old^-EVs treatment groups 4 weeks after surgery, n = 4. Data are presented as the mean ± SD (*: P < 0.05; **: P < 0.01; ***: P < 0.001)

Before starting the therapeutic assays of the EVs treatment, the safety of EV treatment should be noted. As an exogenous and allogeneic bioactive therapy, various previous researches have confirmed its biosafety. By analyzing the histopathological characteristics of the liver/kidney and biochemical examination after EV treatment, it was found that no significant liver or kidney toxicity was detected in any group (Additional file 1: Figure S2 and Additional file 1: Figure S3). This finding demonstrated that both EVs were safe in tendinopathy treatment.EVs-based therapy represents a promising field in regeneration medicine, and the effects of ADMSC-EVs from donors of different ages in tendinopathy remain unknown. One week after tendinopathy was induced in mice with collagenase I injection, both ADMSC^young^-EVs and ADMSC^old^-EVs were used in therapy. An equal volume of PBS injection was adopted as mock treatment. Four weeks later, the tendon tissues in different groups were collected for advanced analyses. As shown in Fig. 4a, the tendon fibers in the tendinopathy group were disordered, with obvious wavy changes in arrangement, incomplete structure, infiltration of inflammatory cells, obvious rounding of nuclei, and a significant increase in cell density. When the therapeutic effects of different EVs were considered, only ADMSC^young^-EVs alleviated the tendinopathy phenotypes. Because pathological fibrosis was involved in the progression of tendinopathy, Masson's trichrome staining was used in fibrosis detection. After ADMSC^young^-EV treatment, abnormal collagen deposition induced by tendinopathy was significantly ameliorated; however, ADMSC^old^-EV therapy failed to improve tendon fibrosis in this study (Fig. 4b). Increased tenocyte apoptotic rates were observed in the tendinopathy model, and ADMSC^young^-EVs, but not ADMSC^old^-EVs, rescued cellular apoptosis after local injection (Fig. 4c). Abnormal extracellular matrix (ECM) remodeling was a key characteristic and pathological progress in tendinopathy, and increased COL I, COL III, MMP3 and MMP9 expression as well as decreased COL III/COL I rate and TIMP-1 content were detected in the tendinopathy mice (Fig. 4d–e). Compared with mock therapy, ADMSC^young^-EV injection, but not ADMSC^old^-EV treatment, was effective in normalizing ECM remodeling. The biomechanical functions were also evaluated, and it was found that the maximum tensile load and stiffness of tendons were significantly depressed in tendinopathy mice. Higher maximum tensile load and stiffness were observed after ADMSC^young^-EV treatment, and no protective effects were observed in the ADMSC^old^-EVs (Fig. 4f). Thus, ADMSC^young^-EVs, but not ADMSC^old^-EVs, alleviated the pathological structural, functional and biomechanical properties in tendinopathy mice.

### ADMSC^young^-EVs, but not ADMSC^old^-EVs, alleviated cell death and cellular senescence of tenocytes treated with IL-1β

**Fig. 5:**
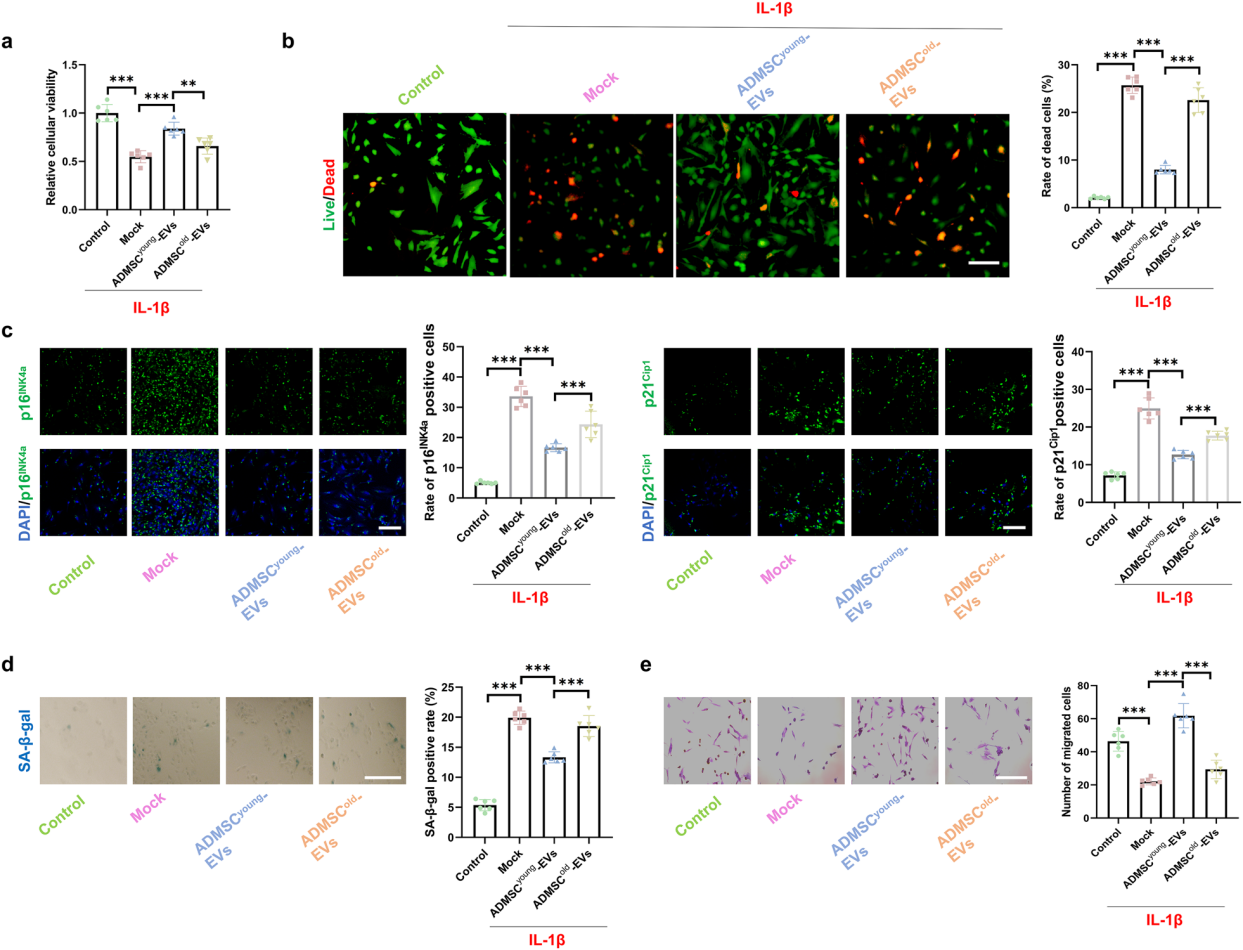
ADMSC^young^-EVs, but not ADMSC^old^-EVs, alleviated cell death and cellular senescence of tenocytes treated with IL-1β. **a** The cellular viability in the control, IL-1β, IL-1β + ADMSC^young^-EVs and IL-1β + ADMSC^old^-EVs tenocyte groups, n = 6. **b** Live/dead assay and rate of dead cells in the control, IL-1β, IL-1β + ADMSC^young^-EVs and IL-1β + ADMSC^old^-EVs tenocytes groups, n = 6. Live cells are marked green, and dead cells are marked red. Scale bar = 50 μm, n = 6. **c** Immunofluorescence staining of p16^INK4A^ (green), p21^CIP1^ (green) and nuclear (DAPI, blue) in the control, IL-1β, IL-1β + ADMSC^young^-EVs and IL-1β + ADMSC^old^-EVs tenocytes groups. Scale bar = 200 μm, n = 6. **d** SA-β-gal activity and the percentages of SA-β-gal-positive cells in ADMSC^young^ and ADMSC^old^. Scale bar = 50 μm, n = 6. **e** Images and quantification of migrated tenocytes in the control, IL-1β, IL-1β + ADMSC^young^-EV and IL-1β + ADMSC^old^-EV groups. Scale bar = 50 μm, n = 6. Data are presented as the mean ± SD (***: P < 0.001)

Tenocytes are a key type of cell influenced by tendinopathy, and IL-1β was adopted to mimic the pathological status and inspect the protective effects of EVs derived from ADMSCs. Pre-treatment of 2.5 × 10^9^ particles/1 × 10^5^ cells of ADMSCyoung-EVs/ADMSCold-EVs were conducted for 24 h and then 2.5 ng/mL IL-1β (Sigma-Aldrich, USA) treatment for 8 h was applied to generate an *in-vitro* tenocytes damage model in each group. Compared with the control group, decreased cellular viability was induced by IL-1β intervention. Protective effects on cellular viability were observed in the ADMSC^young^-EV group but not in the ADMSC^old^-EV treatment group (Fig. 5a). A higher cell death rate was detected in IL-1β-treated tenocytes, and ADMSC^young^-EVs significantly protected tenocytes; however, ADMSC^old^-EVs did not rescue tenocytes treated with IL-1β (Fig. 5b). Cellular senescence is both an important cause and a key pathological characteristic of tendinopathy. Based on immunostaining of cellular senescence markers, including p16^INK4A^ and p21^CIP1^, and SA-β-gal staining, 100 ng/ml IL-1β significantly induced cellular senescence, and pathological senescence was alleviated by ADMSC^young^-EVs but not ADMSC^old^-EVs (Fig. 5c–d). The migration ability was detected with a transwell assay. As shown in Fig. 5e, only ADMSC^young^-EV intervention restored the impaired cellular migration ability of tenocytes induced by IL-1β (P < 0.001).

### ADMSC^young^-EVs, but not ADMSC.^old^-EVs, alleviated pathological ECM remodeling of tenocytes induced by TGF-β1

**Fig. 6:**
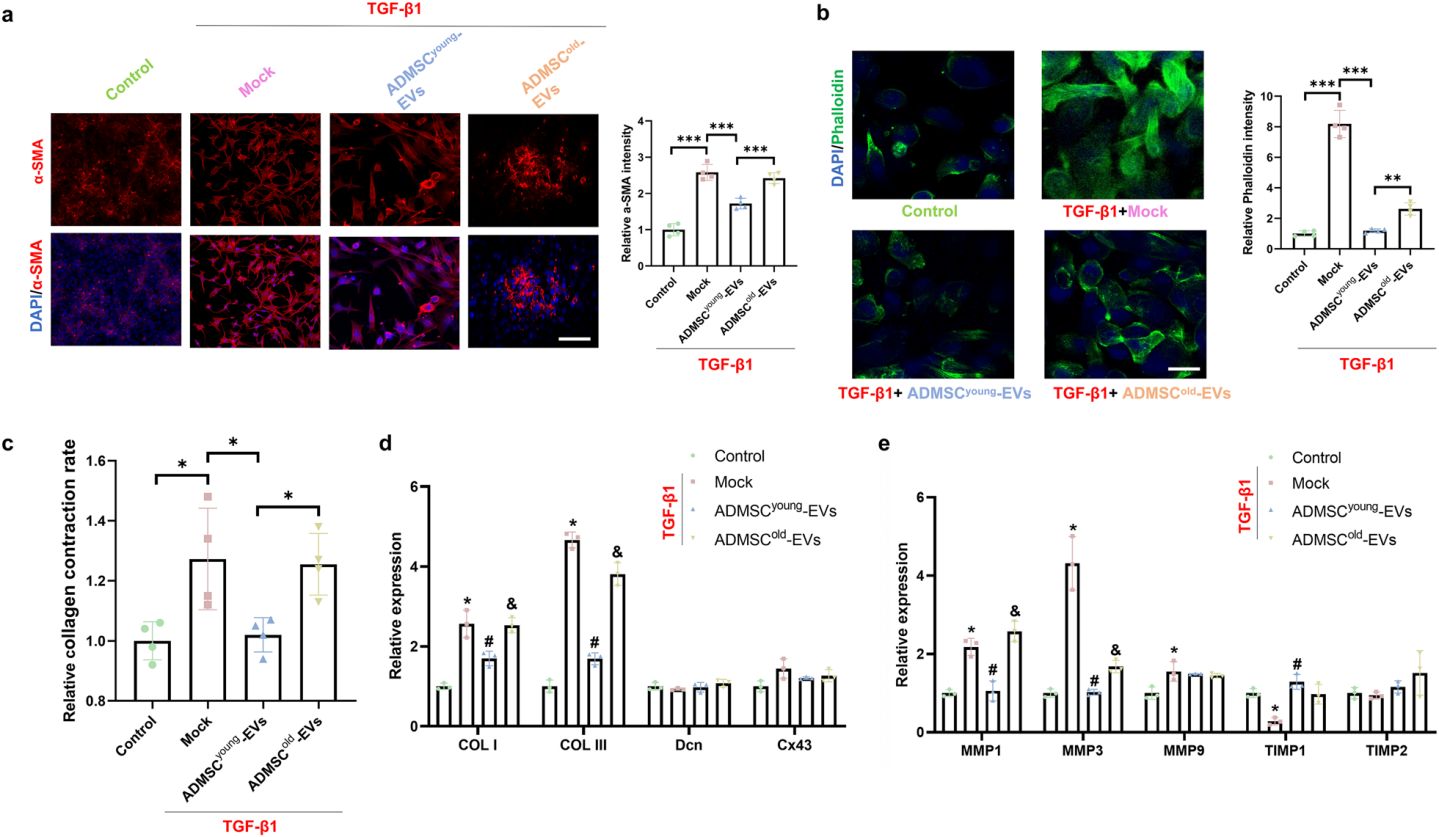
ADMSC^young^-EVs, but not ADMSC^old^-EVs, alleviated pathological ECM remodeling of tenocytes induced by TGF-β1. **a** Immunofluorescence staining and relative intensity of α-SMA (red) and nuclei (DAPI, blue) in the control, TGF-β1, TGF-β1 + ADMSC^young^-EV and TGF-β1 + ADMSC^old^-EV tenocyte groups. Scale bar = 200 μm, n = 4. **b** Immunofluorescence staining and relative intensity of phalloidin (green) and nuclei (DAPI, blue) in the control, TGF-β1, TGF-β1 + ADMSC^young^-EV and TGF-β1 + ADMSC^old^-EV tenocyte groups. Scale bar = 200 μm, n = 4. **c** Relative collagen contraction rate in the control, TGF-β1, TGF-β1 + ADMSC^young^-EV and TGF-β1 + ADMSC^old^-EV tenocyte groups, n = 4. **d**–**e** Relative expression of collagen formation- and degradation-related genes in the control, TGF-β1, TGF-β1 + ADMSC^young^-EV and TGF-β1 + ADMSC^old^-EV tenocyte groups, n = 3. Data are presented as the mean ± SD (*: control vs. TGF-β1 treatment; #: TGF-β1 treatment vs. TGF-β1 + ADMSC^young^-EVs treatment; &: TGF-β1 + ADMSC^young^-EVs vs. TGF-β1 + ADMSC^old^-EVs treatment groups, P < 0.05)

Abnormal ECM remodeling during tendinopathy is caused by various growth factors, in which TGF-β1 is regarded as a key factor. Pre-treatment of 10 ng/mL of TGF-β1 for 24 h was conducted to induce a pathological profibrotic status and then the culture medium would be replaced with the complete medium. Then 2.5 × 10^9^ particles/1 × 10^5^ cells of ADMSCyoung-EVs or ADMSCold-EVs treatment for 24 h would be conducted before advanced cellular experiments. As shown in Fig. 6a, upregulated α-SMA in tenocytes was induced by TGF-β1 and ADMSC^young^-EVs but not ADMSC^old^-EVs and significantly reduced the expression of α-SMA in the TGF-β1-treated in vitro model. Phalloidin staining was also adopted for actin imaging, and both EVs were effective in actin expression regulation; however, a better therapeutic effect was detected in the ADMSC^young^-EV group than in the ADMSC^old^-EV group (Fig. 6b). The collagen contraction rates were detected among all the groups and demonstrated that ADMSC^young^-EVs, but not ADMSC^old^-EVs, were effective in collagen contraction of tenocytes induced by TGF-β1 (Fig. 6c). Collagen formation (COL I, COL III, Den and Cx43)- and ECM remodeling (MMP1, MMP3, MMP9, TIMP1 and TIMP2)-related key genes were analyzed among different cell groups. As shown in Fig. 6d–e, abnormal expression levels of COL I, COL III, MMP1, MMP3, MMP9 and TIMP1 were evaluated (P < 0.05). Advanced intervention experiments showed that ADMSC^young^-EVs restored the expression of COL I, COL III, MMP1, MMP3 and TIMP1 (P < 0.05); however, no significant effects were detected in the ADMSC^old^-EV treatment group (P > 0.05).

### ADMSC^young^-EVs protected tenocytes treated with IL-1β by restoring NAD + biosynthesis and the NAMPT/SIRT1/PPARγ/PGC-1α pathway

**Fig. 7:**
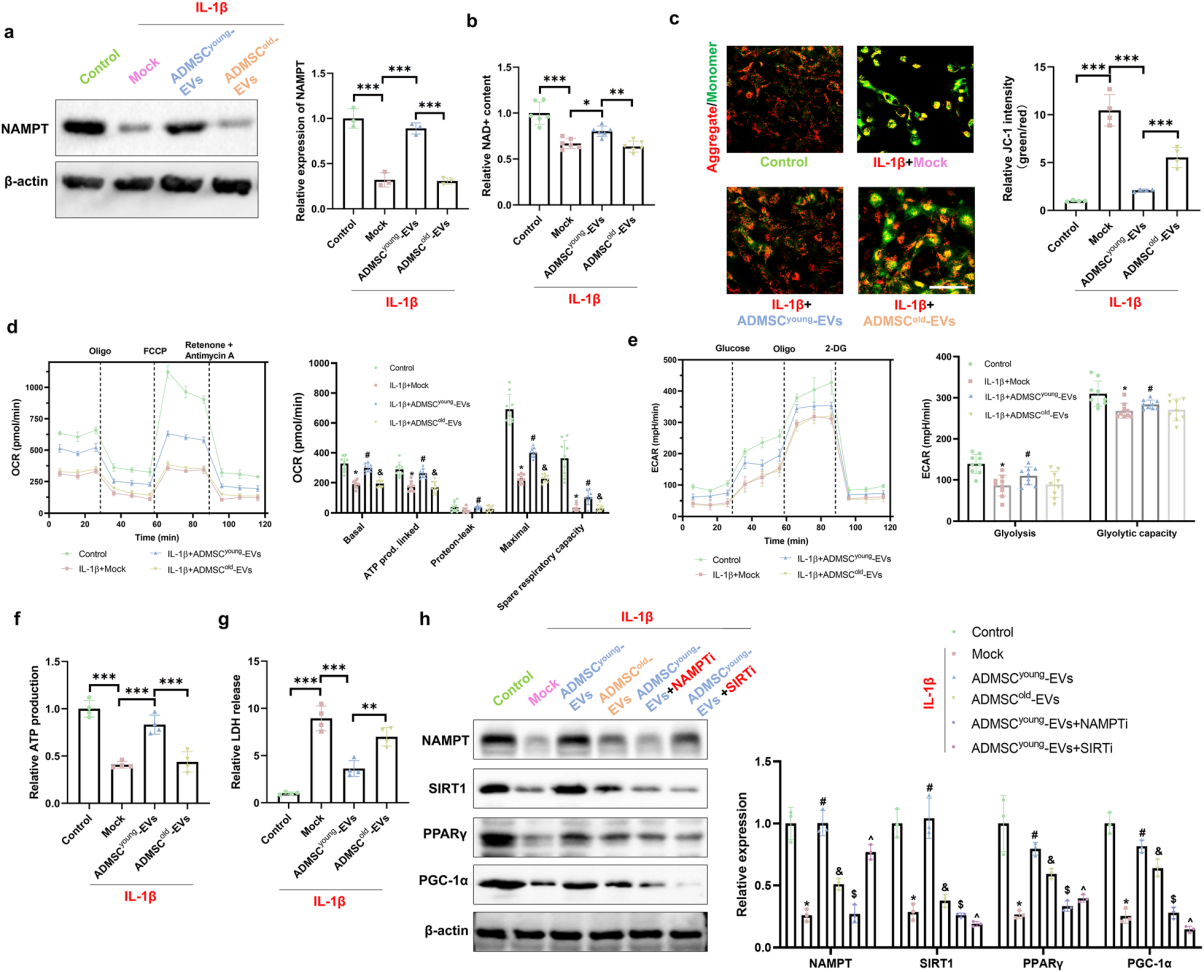
ADMSC^young^-EVs protected tenocytes treated with IL-1β by restoring NAD + biosynthesis and the NAMPT/SIRT1/PPARγ/PGC-1α pathway. **a** Western blotting and quantification of NAMPT in the control, IL-1β, IL-1β + ADMSC^young^-EV and IL-1β + ADMSC^old^-EV tenocyte groups, n = 3. **b** Relative NAD+ content in the control, IL-1β, IL-1β + ADMSC^young^-EV and IL-1β + ADMSC^old^-EV tenocyte groups, n = 4. **c** JC-1 staining of tenocytes in the control, IL-1β, IL-1β + ADMSC^young^-EVs and IL-1β + ADMSC^old^-EV groups. Aggregates are shown in red, and monomers are shown in green. The relative JC-1 intensity was quantified, and n = 4. Scale bar = 200 μm. **d** OCR curves and OCR respiratory reactions in the control, IL-1β, IL-1β + ADMSC^young^-EV and IL-1β + ADMSC^old^-EV tenocyte groups. The basal OCR, ATP production, protein leak, maximal OCR, and spare respiratory capacity were calculated, n = 4. **e** The ECAR curves and ECAR respiratory reactions in the control, IL-1β, IL-1β + ADMSC^young^-EV and IL-1β + ADMSC^old^-EV tenocyte groups, n = 4. The gliosis and glycolytic capacity were calculated. **f** The relative ATP production ability in the control, IL-1β, IL-1β + ADMSC^young^-EV and IL-1β + ADMSC^old^-EV tenocyte groups, n = 4. **g** Relative LDH release in the control, IL-1β, IL-1β + ADMSC^young^-EV and IL-1β + ADMSC^old^-EV tenocyte groups, n = 4. **h** Western blotting and quantification of NAMPT, SIRT1, PPARγ and PGC-1α in the control, IL-1β, IL-1β + ADMSC^young^-EVs, IL-1β + ADMSC^old^-EVs, IL-1β + ADMSC^young^-EVs + NAMPT inhibitor and IL-1β + ADMSC^young^-EVs + SIRT1 inhibitor tenocyte groups, n = 3. (*: control vs. IL-1β treatment; #: IL-1β treatment vs. IL-1β + ADMSC^young^-EVs treatment; &: IL-1β + ADMSC^young^-EVs vs. IL-1β + ADMSC^old^-EVs treatment groups, $: IL-1β + ADMSC^young^-EVs vs. IL-1β + ADMSC^young^-EVs + NAMPT inhibitor; ^: IL-1β + ADMSC^young^-EVs vs. IL-1β + ADMSC^young^-EVs + SIRT1 inhibitor, P < 0.05; **: P < 0.01; ***: P < 0.001)

EVs provided potential beneficial effects through their cargos, and the rehibition effects were analyzed in advanced experiments. Considering that NAMPT was enriched in ADMSC^young^-EVs compared to ADMSC^old^-EVs, the effects of ADMSC^young^-EVs on NAD+ metabolism and subsequent signaling activation were investigated. As shown in Fig. 7a, b, decreased NAMPT expression and NAD+ content in tenocytes were induced by IL-1β treatment. By analyzing the effects of ADMSC-derived EVs on NAMPT expression and NAD+ synthesis, only the young EVs improved NAMPT expression to increase NAD+ content. Considering the key roles of NAD+ in mitochondrial function, the mitochondrial membrane permeability (MMP), respiratory function and ATP biogenesis effect were analyzed. According to the data presented in Fig. 7c, both types of EVs were protective in MMP maintenance; however, ADMSC^young^-EVs demonstrated a better effect. Mitochondria are the centers of cellular energy metabolism; thus, both the ECAR and OCR were adopted to analyze cellular oxidative phosphorylation and glycolytic capacity. As shown in Fig. 7d, e, ADMSC^young^-EV treatment alleviated basal OCR, ATP production, protein leakage, maximal OCR, spare respiratory capacity, gliosis and glycolytic capacity damage induced by IL-1β treatment. However, no significant protective effects were detected with ADMSC^old^-EV treatment (P > 0.05). Cellular ATP contents were also considered, and the impaired ATP production induced by IL-1β was alleviated by ADMSC^young^-EV treatment (P < 0.001); however, a poorer protective effect was detected in the ADMSC^old^-EVs than in the ADMSC^young^-EVs (Fig. 7f, P < 0.001). LDH release was also evaluated, and it was found that both ADMSC^young^-EVs and ADMSC^old^-EVs could alleviate LDH release caused by IL-1β treatment (P < 0.05). In addition, ADMSC^young^-EVs demonstrated a better effect in LDH release improvement than the ADMSC^old^-EVs (Fig. 7g, P = 0.001). ADMSC^young^-EVs demonstrated an upregulation of NAMPT, and higher NAD + contents provided more substrates for SIRT1 function. As presented in Fig. 7h, a significant decrease in the NAMPT/SIRT1/PPARγ/PGC-1α pathway was detected in tenocytes treated with IL-1β. Advanced intervention experiments showed that ADMSC^young^-EVs, but not ADMSC^old^-EVs, upregulated the signaling pathway. Considering the key role of NAMPT and SIRT1 in the protective effects of ADMSC^young^-EVs, inhibition of NAMPT and SIRT1 pretreatment significantly eliminated the regulated signaling pathway.

### ADMSC^young^-EVs, but not ADMSC^old−^EVs, alleviated cellular senescence and promoted M2 polarization in macrophages treated with IL-1β

**Fig. 8:**
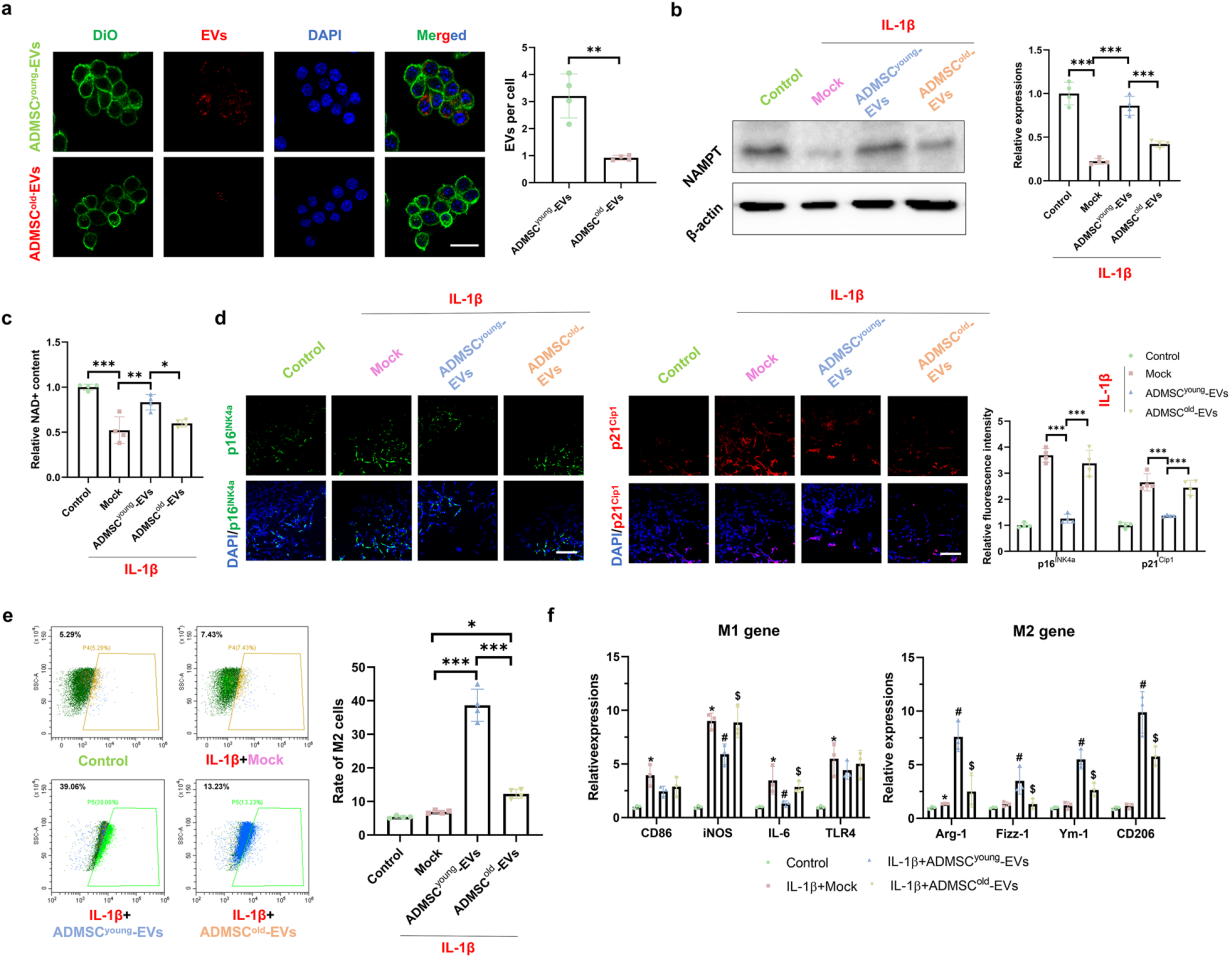
ADMSC^young^-EVs, but not ADMSC^old−^EVs, alleviated cellular senescence and promoted M2 polarization in macrophages treated with IL-1β. **a** The uptake assay of ADMSC^young^-EVs and ADMSC^old^-EVs in cultured macrophages. The cell membranes (labeled with DIO, green), EVs (labeled with DII, red) and nuclei (DAPI, blue) are listed. The uptaken EVs per cell were analyzed. Scale bar = 100 μm and n = 4. **b** Western blotting and quantification of NAMPT in the control, IL-1β, IL-1β + ADMSC^young^-EVs and IL-1β + ADMSC^old^-EVs macrophage groups, n = 3. **c** Relative NAD + content in the control, IL-1β, IL-1β + ADMSC^young^-EV and IL-1β + ADMSC^old^-EV macrophage groups, n = 4. **d** Immunofluorescence staining of p16^INK4A^ (green), p21^CIP1^ (red) and nuclear (DAPI, blue) in the control, IL-1β, IL-1β + ADMSC^young^-EVs and IL-1β + ADMSC^old^-EVs macrophage groups. Scale bar = 200 μm and n = 6. **e** The rate of M2 macrophages in the control, IL-1β, IL-1β + ADMSC^young^-EVs and IL-1β + ADMSC^old^-EVs macrophage groups, n = 4. **f** The relative expression of M1 markers, including CD86, iNOS, IL-6 and TLR4, and M2 markers, including Arg-1, Fizz-1, Ym-1 and CD206, in the control, IL-1β, IL-1β + ADMSC^young^-EV and IL-1β + ADMSC^old^-EV macrophage groups, n = 3. (*: control vs. IL-1β treatment; #: IL-1β treatment vs. IL-1β + ADMSC^young^-EVs treatment; &: IL-1β + ADMSC^young^-EVs vs. IL-1β + ADMSC^old^-EVs treatment groups, P < 0.05; *: P < 0.05; **: P < 0.01 and ***: P < 0.001)

As macrophages are also involved in the progression of tendinopathy, advanced experiments were conducted to explore the roles of ADMSC-derived EVs on macrophages treated with IL-1β. Pre-treatment of 2.0 × 10^9^ particles/1 × 10^5^ cells ADMSCyoung-EVs/ADMSCold-EVs in cultured macrophages were conducted for 24 h and then 10 ng/mL IL-1β treatment for 48 h was applied to generate an *in-vitro* inflammatory stress conditions in each group. A total of 10^8^/ml DII iodide particles were used in the EV uptake assay, and it was found that ADMSC^young^-EVs demonstrated a better uptake rate than the ADMSC^old^-EVs (P = 0.001, Fig. 8a). To demonstrate the effects of EVs derived from ADMSCs on the expression of NAMPT and NAD+ production in IL-1β-treated macrophages, it was found that only ADMSC^young^-EVs could significantly promote NAMPT expression and NAD+ production in IL-1β-treated macrophages (P < 0.001, Fig. 8b, c). In addition, the expression of cellular senescence-related markers, including p16^INK4A^ and p21^CIP1^, in different groups was assessed, and it was found that ADMSC^young^-EVs (P < 0.05), but not ADMSC^old^-EVs, alleviated the cellular senescence-related markers in macrophages treated with IL-1β (P > 0.05, Fig. 8d). The rate of M2 polarization, as well as the expression of M1/M2 markers, was detected using flow cytometry. As shown in Fig. 8e, higher M2 rates were detected in both EV-treated groups, and a higher M2 rate was detected in the ADMSC^young^-EV group than in the ADMSC^old^-EV group (P < 0.001). When the expression of M1/M2 markers was considered, ADMSC^young^-EVs, but not ADMSC^old^-EVs, significantly downregulated M1 markers and upregulated M2 markers (Fig. 8f).

### ADMSC^young^-EVs regulated the NAMPT/SIRT1/Nf-κb p65/NLRP3 pathway in macrophages and alleviated the in vitro tendinopathy model through direct and indirect pathways

**Fig. 9:**
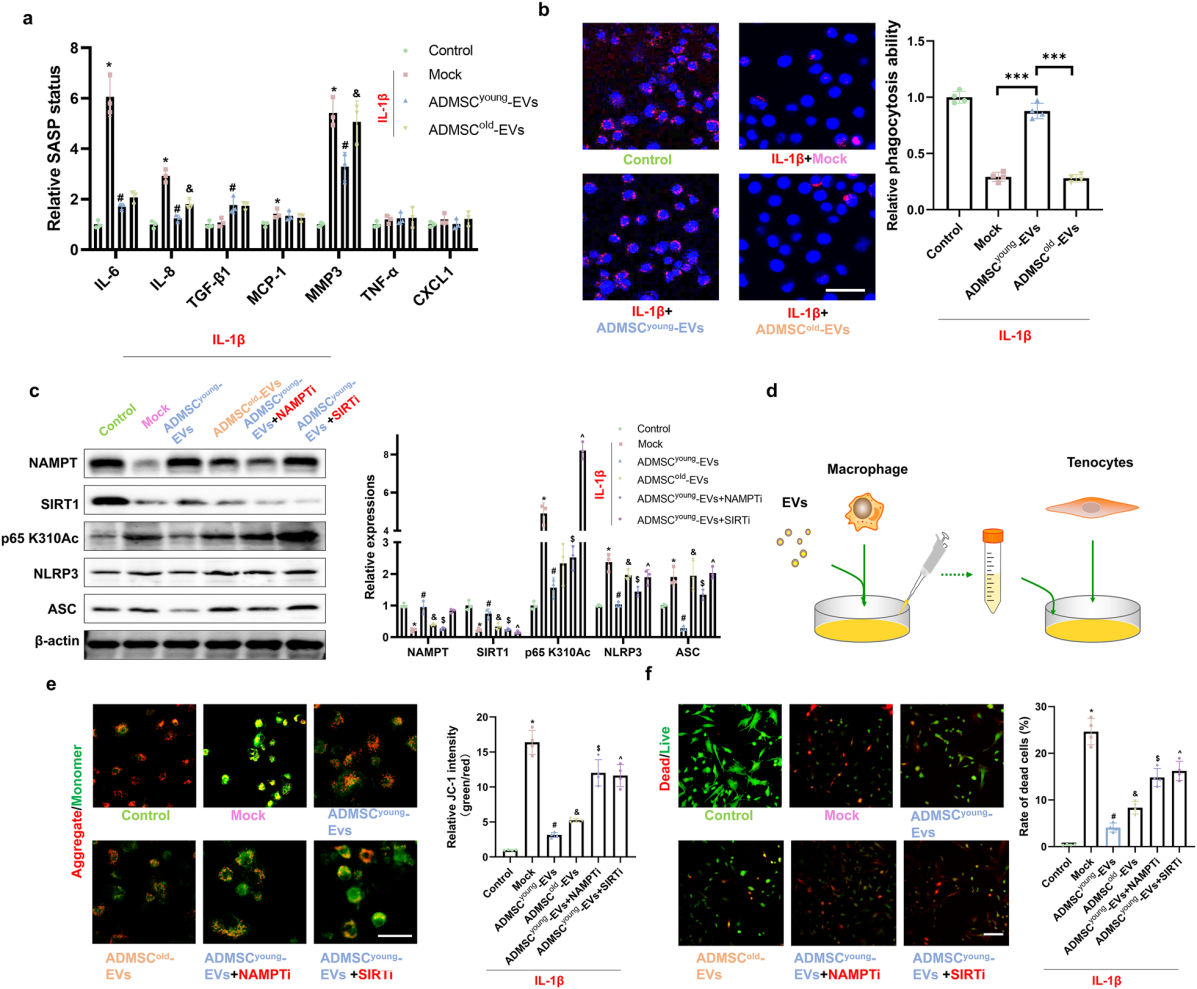
ADMSC^young^-EVs regulated the NAMPT/SIRT1/Nf-κb p65/NLRP3 pathway in macrophages and alleviated tendinopathy in vitro through direct and indirect pathways. **a** The relative SASP status, including IL-6, IL-8, TGF-β1, MCP-1, MMP3, TNF-α and CXCL1 in the control, IL-1β, IL-1β + ADMSC^young^-EV and IL-1β + ADMSC^old^-EV macrophage groups, n = 3. **b** The relative phagocytosis ability in the control, IL-1β, IL-1β + ADMSC^young^-EV and IL-1β + ADMSC^old^-EV macrophage groups. Scale bar = 50 μm and n = 4. **c** Western blotting and quantification of NAMPT, SIRT1, p65 Nf-κb K310Ac, NLRP3 and ASC in the control, IL-1β, IL-1β + ADMSC^young^-EV, IL-1β + ADMSC^old^-EV, IL-1β + ADMSC^young^-EVs + NAMPT inhibitor and IL-1β + ADMSC^young^-EVs + SIRT1 inhibitor macrophage groups, n = 3. **d** A schematic diagram of the indirect contact macrophage-tenocyte crosstalk. **e** JC-1 staining of tenocytes in the control, IL-1β-treated macrophage medium, IL-1β-treated macrophage medium + ADMSC^young^-EV and IL-1β-treated macrophage medium + ADMSC^old^-EV groups and the IL-1β-treated macrophage medium + ADMSC^young^-EVs + NAMPT inhibitor and IL-1β-treated macrophage medium + ADMSC^young^-EVs + SIRT1 inhibitor groups. Aggregates are shown in red, and monomers are shown in green. The relative JC-1 intensity was quantified, and n = 4. Scale bar = 50 μm. **f** Live/dead assay of tenocytes in the control, IL-1β-treated macrophage medium, IL-1β-treated macrophage medium + ADMSC^young^-EV, IL-1β-treated macrophage medium + ADMSC^old^-EV, IL-1β-treated macrophage medium + ADMSC^young^-EVs + NAMPT inhibitor and IL-1β-treated macrophage medium + ADMSC^young^-EVs + SIRT1 inhibitor groups. Live cells are indicated in green, and dead cells are indicated in red. The rate of cell death was quantified. Scale bar = 100 μm and n = 4. (*: control vs. IL-1β treatment; #: IL-1β treatment (or treated macrophage medium) vs. IL-1β (or treated macrophage medium) + ADMSC^young^-EV treatment; &: IL-1β (or treated macrophage medium) + ADMSC^young^-EV vs. IL-1β (or treated macrophage medium) + ADMSC^old^-EV treatment groups, $: IL-1β (or treated macrophage medium) + ADMSC^young^-EVs vs. IL-1β (or treated macrophage medium) + ADMSC^young^-EVs + NAMPT inhibitor; ^: IL-1β (or treated macrophage medium) + ADMSC^young^-EVs vs. IL-1β (or treated macrophage medium) + ADMSC^young^-EVs + SIRT1 inhibitor, P < 0.05; **: P < 0.01; ***: P < 0.001)

Macrophages can regulate the local inflammatory microenvironment and influence the biological functions of tenocytes. By detecting the expression of SASP markers in IL-1β-treated macrophages, increased expression levels of IL-6, IL-8, MCP-1 and MMP3 were detected. ADMSC^young^-EV treatment significantly depressed the expression of IL-6, IL-8, MCP-1 and MMP3; however, no significant effect in the ADMSC^old^-EV treatment group was detected (Fig. 9a). Interestingly, ADMSC^young^-EVs upregulated the expression of TGF-β1 in macrophages. In addition, the phagocytosis effects in different groups were analyzed, and it was found that IL-1β treatments significantly impaired the phagocytosis effects in macrophages. When the detailed effects of EV treatments were considered, it was found that ADMSC^young^-EVs, but not ADMSC^old^-EVs, could restore the phagocytosis effects (Fig. 9b). When the detailed molecular mechanism was considered, IL-1β treatment depressed the expression of NANPT and SIRT1 and subsequently promoted p65 Nf-κb K310Ac, NLRP3 and ASC expression. Advanced EV treatments showed that ADMSC^young^-EVs could significantly relieve pathological signaling, and these effects were blocked by NAMPT and SIRT1 inhibitor pretreatment (Fig. 9c).

To detect the indirect effects of macrophage protection on tenocyte biological effects, a cell culture medium transfer method was generated (Fig. 9d). The culture media of 6 groups, including the control group, 100 ng/ml IL-1β-treated macrophages, 100 ng/ml IL-1β + ADMSC^young^-EVs-treated macrophages, 100 ng/ml IL-1β + ADMSC^old^-EV-treated macrophages and NAMPT inhibitor/SIRT1 inhibitor pretreatment + 100 ng/ml IL-1β + ADMSC^young^-EV-treated macrophages, were generated. After 24 h, the old culture media were replaced with fresh media, and the media were used after 48 h of culture. Media of macrophages were mixed with culture media of tenocytes at a volume rate of 1:1.5. After 24 h of cell culture medium transfer, both JC-1 staining and live/death assays were conducted. As shown in Fig. 9e, f, culture media from IL-1β-treated macrophages demonstrated an impaired MMP and increased cell death rate (P < 0.001). In addition, ADMSC^young^-EV treatment, but not ADMSC^old^-EV treatment, in IL-1β-treated macrophages demonstrated a protective effect in cultured tenocytes. The data from both the NAMPT inhibitor and SIRT1 inhibitor groups showed that NMAPT and SIRT1 demonstrated a key role in macrophage-tenocyte crosstalk. As SIRT1 was a key regulated gene and the expression of SIRT1 in ADMSC^young^-EV and ADMSC^old^-EV by western blot showed that SIRT1 was not a transport in the EVs derived from ADMSCs (Additional file 1: Figure S4).

## Discussion

In this study, a novel mechanism and interesting findings demonstrated that ADMSC^young^-EVs, but not ADMSC^old^-EVs, provided therapeutic effects for tendinopathy in a "One-Stone-Two-Birds" manner. Compared with ADMSC^old^-EVs, one key biosynthesis enzyme in NAD+ metabolism, NAMPT, was more abundant in ADMSC^young^-EVs. In addition, NAMPT is a key functional cargo that protects both tenocytes from cell death and macrophages from inflammatory activation. This study highlights the potential of the EVs derived from ADMSCs in the management of tendinopathy.

The treatments for tendinopathy remain quite limited, however, novel therapies, including platelet-rich plasma, stem cells and biomaterials [24–26], insufficient effects and potential harm exists in these therapies. EVs therapy is an advanced cell-free therapy and are reported to be a promising operation for incurable disorders. EVs are derived from several different MSC sources, including bone marrow, infrapatellar fat pads and amniotic membranes [27–29]. ADMSC-derived EVs were reported to promote the healing of traumatized Achilles tendons, which demonstrated that they may be beneficial for tendinopathy recovery [12, 30]. The results of this study provide supportive data for ADMSC^young^-EVs in tendinopathy therapy and highlight their effects on both tenocytes and macrophages. Two previous studies on ADMSC-derived EVs in tendon repair did not provide the age of donors [31, 32]; however, it was natural to conjecture that no ADMSCs from aged mice or humans were used in their studies. Thus, our data were consistent with previous studies, and additional knowledge was provided for future studies.

ADMSCs are regarded as promising seed cells for EV production because of convenience in clinical ethics, the abundant sources and stable genomics. Both autologous and allogeneic adipose tissues could be used in ADMSC isolation, and the autologous tissues were more. Considering that tendinopathy is an age-related degenerative disorder, the old ages of donors are a potential barrier to standardize EVs and guarantee their therapeutic efficacy. A key obstacle to the clinical use of EVs is the difficulty of generating EVs, such as by drugs, because of the various influencing factors of EV characteristics. Age was one of the most common interfering factors, and EVs derived from cells or organs from older donors failed to produce equal threptic effects. In a recent elegant study, it was found that young osteocyte-derived EVs (OCY^young^-EVs) benefited Alzheimer's disease; however, the protective effects were diminished by OCY^old^-EV treatment [33]. There was a general information that sexual maturity of mice is at about 35 days (5 weeks) while 8 weeks aged mice were mostly frequently used in animal experiments. In the aging related researches, 2 months aged mice, which was regarded as less than 20 years old in human, were generally used in pathological mechanism detection [34, 35]. Considering that a previous study focusing on extracellular NAMPT in EVs from adipose tissue used mice of 4 months old as the young group, we used the mice in 2 months old in this current study to provide more knowledge in this field. To our knowledge, this was the first study regarding the effects of aging on the function of EVs in tendinopathy treatment. Compared with ADMSC^young^-EVs, ADMSC^old^-EVs failed to protect against tendinopathy in both in vivo and in vitro studies.

When comparing the basic characteristics of ADMSC^young^-EVs and ADMSC^old^-EVs, it was found that they were in a relatively closed particle size and surface potential and it was found that EV-derived cellular donors at different ages demonstrated similar exosomal characteristics. There were also several differences between the two EVs. A key point was a relatively higher EV yield in the ADMSC^old^, and this could be explained by the chronic inflammatory stress in senescent MSCs. Because the key characteristic of senescent cells was the abnormal secretory capacity, increased EVs production could explain the aging-related microenvironment, at least in part. In addition, it was found that ADMSC^young^-EVs were more easily internalized by macrophages in in vitro experiments. A higher uptake rate could help ADMSC^young^-EVs produce a better protective effect. There were compressive differences in EV size, cargos and biological effects, which guided their application in the clinic, as summarized by a recent review [18]. In the analyses of NAD+ metabolism-related factors, key genes were dysregulated, except for NAMPT. For instance, higher SIRT1 content was detected in young ADMSCs; however, the SIRT1 extract content in different EVs was not shown in this study. As a well-accepted longevity-related factor as well as an NAD+ consumer, the role of EVs in SIRT1-related cellular consumption would be a fascinating topic. Of note, only adipose tissues on the inguinal, which is white adipose tissue, were used in this study. In the clinic, subcutaneous fat, which is also white adipose tissue, was used in ADMSC isolation, and our data were closely related to clinical translation. Of note, there was another type of adipose tissue, brown adipose, and it produced quite different characteristics, including ASMSC generation and EVs production [36]. Advanced experiments on this issue should be performed to clarify the influence of adipose donors on biological effects.

Impaired NAD+ metabolism dysfunction and decreased NAD+ content was detected in the ADMSC^old^, and one rate-limiting enzyme, NAMPT, was adopted in advanced experiments. A previous groundbreaking study showed that circulating eNAMPT levels were significantly decreased with age in mice and humans, and adipose-specific overexpression of NAMPT increased circulating eNAMPT levels, thus leading to increased NAD+ levels in aged mice [23]. Another interesting finding was that circulating eNAMPT was carried by EVs, thus highlighting the potential regulatory pathway of aging-associated EVs derived from adipose-degenerative diseases. Our study focused on ADMSCs and reported that significantly low NAMPT content was detected in old ADMSCs. Advanced experiments are required to investigate the complex relationship between aging, cellular senescence of ADMSCs and adipose tissue aging [37]. A total of two target cells, tenocytes and macrophages, were studied in this work. Because tenocytes are a key cellular component of tendon tissue, the protective effects of ADMSC^young^-EVs induced overexpression of NAMPT, and subsequent increased production of NAD+ promoted the survival of tenocytes treated with IL-1β. In the detailed molecular mechanism study, mitochondrial function and the NAMPT/SIRT1/PPARγ/PGC-1α pathway were identified in this study. Because NAD+ is a key metabolic coenzyme in mitochondrial function, NAMPT activation is regarded as a promising target for mitochondrial protection [38]. Peroxisome proliferator-activated receptor gamma (PPARγ) is a key regulator of adipogenesis and adipose tissue development. A previous study focused on the effects of NAMPT on alleviating atherosclerosis by regulating PPARγ pathway-based monocyte differentiation [39]. Consistent with a previous study, NAMPT activation-related PPARγ function was related to improved function in tenocytes. Macrophages were the focus of this study, and the NAMPT/SIRT/Nf-κb p65 pathway was identified as the key signaling pathway. A previous study reported that in vitro EV-educated macrophages demonstrated an M2-like phenotype, and thus, educated macrophage treatment demonstrated an advanced therapeutic strategy [40]. In this study, NAMPT upregulation and increased NAD+ contents related to ADMSC^young^-EV treatments promoted M2 polarization. Due to the anti-inflammatory effects and pro-regenerative characteristics of M2 macrophages, a “One-Stone-Two-Birds” pattern was generated.

The crosstalk between macrophages and tenocytes could be regulated by EV treatment, and it was found that the culture medium of macrophages with different EV treatments influenced the biological effects of tenocytes in this study. It was found that NAMPT and SIRT in macrophages were key factors in macrophage-tenocyte crosstalk. It was found that ADMSC^young^-EVs significantly alleviated cellular senescence-related SASP and phagocytosis. Because tenocytes function in a microenvironment, the inflammatory environment is influenced by macrophage polarization. Because macrophages are a key cellular contributor to inflammatory responses, our results showed that ADMSC^young^-EV treatment significantly alleviated SASP status and relieved inflamm-aging, slowing down the process of tendinopathy. Phagocytosis is another key function of macrophages and is important in damaged tissue clearance and organ damage repair [41]. In an inflammatory bowel disease study, it was found that NAMPT could mitigate the inflammatory severity by promoting phagocytosis in inflammatory macrophages [42]. In this study, our data showed that ADMSC^young^-EVs with NAMPT could effectively promote macrophage phagocytosis. Further experiments on the contribution of regulated phagocytosis in alleviating the progression of tendinopathy are needed.

## Conclusions

In summary, our data support that ADMSC^young^-EVs contribute as a potential therapy for tendinopathy. NAMPT was significantly increased in ADMSC^young^-EVs, and NAMPT regulation and subsequent NAD+ production in both tenocytes and macrophages protected against tendinopathy. Because ADMSC^young^-EVs demonstrated a “One-Stone-Two-Birds” effect, a promising therapy was generated to include the age in donor selection during ADMSC-derived EV production.

## Methods

### Animals and treatments

The procedures of animal experiments were approved by the Institutional Animal Care and Use Committee of Tongji University. Young (2 months, weight 20–25 g) and old (22 months, weight 32–40 g) C57BL/6 J mice were obtained from Vital River Laboratory Animal Technology Co., Ltd. (Beijing, China). At least one week of acclimation was allowed before advanced experiments. The mice were raised under specific pathogen-free (SPF) conditions with a 12 h light/dark cycle at a temperature of 18–22 °C and humidity of 50–60%. Both male and female mice were used in the ADMSC isolation, while only male mice were used in tendinopathy related animal experiments. A total of four groups of mice, including the control, tendinopathy, tendinopathy with ADMSCyoung-EVs and tendinopathy with ADMSCold-EVs treatment groups, were involved in this study and 12 mice were included in each group. To induce a tendinopathy phenotype, 20 μL of 1% type I collagenase (Sigma‒Aldrich, USA) was injected around the Achilles tendon. For EV treatments, EVs at a concentration of 10^9^ particles/20 μL local injection were administered once after tendinopathy induction.

### ADMSC isolation and EVs production

ADMSC isolation and primary culture were conducted from both young and old C57BL/6 mice as described previously [9, 43]. Briefly, both young and old mice were sacrificed, and the adipose tissues on the inguinal were collected for ASMSC isolation. The adipose tissues were digested with 1 mg/mL collagenase I at 37 °C for 30 min. The cell suspensions were filtrated and then centrifuged at 1200 r/min for 7 min. The pellets were resuspended, and ADMSCs were cultured with DMEM/F12 1:1 medium (Gibco, Thermo Fisher Scientific, USA) containing 15% fetal bovine serum (FBS, Gibco, Thermo Fisher Scientific, USA). ADMSCs from young or old mice were recorded as ADMSC^young^ or ADMSC^old^, respectively, and ADMSC of the passage four was used in advanced in vitro experiments. The cultured ADMSCs were collected, digested with trypsin and then washed 3 times with PBS. After adjusting the cell suspensions to approximately 1 × 10^6^ cells/ml, the cell suspensions were centrifuged at 1500 r/min for 5 min and then washed with PBS. A total of 100 μl of PBS was added to generate a cellular sample and 10 μl of CD29 (Cat.No. 11-0291-82, Thermo Fisher Scientific, USA), CD90 (Cat.No. 11-0900-81, Thermo Fisher Scientific, USA) and CD45 (Cat.No. 11-0451-82, Thermo Fisher Scientific, USA) primary antibodies were added. After being incubated at room temperature for 20 min and washed with PBS to remove the the unconjugated antibodies, 500 μl of PBS was used to resuspend the suspensions for flow cytometry identification in an LSRII flow cytometer (BD Biosciences) and analyses with FlowJo software. EVs production and characterization were conducted following our previous protocol [44]. The culture supernatants of the ADMSCs were collected and gently centrifuged to remove cells and cellular debris. The supernatants were centrifuged at 100,000 × g at 4 °C for 2 h, and the pellets were then resuspended in PBS. After isolation, ADMSC-derived EVs were obtained and stored at − 80 °C until use. A total of 10 μL of EVs was dropped on copper grids, and the morphology of EVs was observed with a transmission electron microscope (Tecnai Spirit, USA). EVs were diluted into 200 μL, and the diameter distributions and zeta potentials were detected using a dynamic light scattering (DLS, Malvern Instruments, UK). The amounts of EVs were measured with nanoparticle tracking analysis (NTA, Particle Metrix, Meerbusch, Germany). Exosomal markers, including Alix (Cat.No. 12422-1-AP, Proteintech, USA) and CD9 (Cat.No. 14025-1-AP, Proteintech, USA), were imaged by immunofluorescence in ADMSC^young^-EVs and ADMSC^old^-EVs. The EVs were fluorescently labelled by incubation with CD9 and Alix antibodies respectively. Fluorescence images of EVs were obtained from Nikon A1 plus multiphoton laser scanning confocal microscopy. Protein was extracted from EVs using RIPA buffer and sonicated at 4 °C. The expression levels of EV markers, including CD9, CD63 (Cat.No. PA5-100713, Thermo Fisher Scientific, USA) and Alix, as well as NAMPT (Cat.No. 11776-1-AP, Proteintech, USA) were analyzed by western blotting. The detailed operations of western blot were shown below.

### Safety assessments

Because both EVs were exogenous and demonstrated potential toxicity, safety assessments were conducted by both observing histological imaging and assessing the physiological function of the liver and kidney. Hematoxylin and eosin (HE) staining was used for histological observation, and biochemistry examinations were used to examine liver and kidney functions. At least 0.75 mL of blood was collected from all mice, and serum samples were obtained for advanced analyses. Blood biochemical analyses were performed at the Shanghai Biological Model Organism Research Center.

### Histological examination and TUNEL staining

The Achilles tendon along with part of the tibia and calcaneus were taken and quickly placed in 4% paraformaldehyde for fixation. The fixed specimens were routinely sectioned and stained. Masson staining was used to image the collagen fiber using a commercial kit (Masson's Trichrome Stain Kit, Solarbio, Beijing, China) according to the manufacturer's instructions. Paraffin-embedded tissue sections were then deparaffinized in xylene and dehydrated in ethanol gradients. The staining was performed according to the instructions of the TUNEL staining kit (Sigma‒Aldrich, USA). Apoptotic cells were positive cells, which were brownish yellow or tan under the light microscope, and non-apoptotic cells were negative cells, which appeared blue.

### Biomechanical testing

For the biomechanical test, freshly isolated tendons were maintained in PBS and stored at − 20 ℃ until use. Both ends of the tendon of the experimental specimen are directly fixed on the instrument to ensure that when the maximum tensile force is reached, the tendon tear site is the tendon or tendon-bone junction rather than the tendon side or the humeral head side falling off the instrument. During the biomechanical experiment, the tensile test was performed on a universal material testing machine (AG-10KNX, Shimadzu, Japan), and the load rate was 0.4 mm/s with a preload of 1 N. The loading load when the tendon was pulled off was observed and recorded in detail and used as the maximum load (N). The stiffness of tendon tissues (force required per mm displacement) was analyzed based on the force/elongation curve.

### ELISA

The concentrations of Col I (Cat. No. KL-ColI-Mu, Kanglang Bioengineering, Shanghai, China), Col III (Cat. No. 02666, Yansheng Bioengineering, Shanghai, China), MMP3 (Cat. No. MMP300, R&D, USA), MMP9 (Cat. No. MMPT90, R&D, USA) and TIMP-1 (Cat. No. MTM100, R&D, USA) in the supernatant from tendon tissue homogenates were measured using commercial ELISA kits (Cat. No. DAB142, DAB140B; R&D, USA). The procedures were conducted following the instructions of the manufacturer. The OD value at 450 nm was detected with a Multiskan Microplate reader (Thermo Fisher Scientific, USA) used for analyses.

### SA-β-gal staining

A senescence β-galactosidase staining kit (Beyotime Biotech, Hangzhou, China) was used to detect cellular senescence in cultured cells. Staining was conducted according to the manufacturer’s instructions. The senescent cells were stained blue, and the percentage of positive.

### NAD+/NADH assay

NAD+ content and the NAD+/NADH ratio were analyzed with an NAD/NADH Assay Kit (Cat. No. ab65348, Abcam; UK). Tissue lysates were used to quantify NAD+ contents and the.

### Cell culture

Primary cultures of tenocytes and macrophages were used for advanced experiments. Briefly, tendon tissue was digested with 1 U/ml dispase (STEMCELL Technologies, USA) and 2 mg/ml collagenase (Worthington Biochemical, USA). Isolated tenocytes were kept in α-MEM (Gibco, USA) containing 10% FBS (Gibco, USA) and type I collagen (Sigma‒Aldrich, USA). After sacrificing the mice, the peritoneal macrophages were isolated and extracted. After 48 h of culture, the morphology of the cells was observed under a microscope and identified by flow cytometry.

A cellular crosstalk system was generated to mimic inflammatory conditions in the tendon microenvironment. Macrophages were treated with IL-1β or EVs, and the culture media were collected to treat the tenocytes. IL-1β pretreatment in macrophages was conducted to induce tendinopathy-related inflammation, and tenocytes were collected for advanced analyses.

### In vitro experiments

IL-1β treatments in tenocytes and macrophages were used to induce an inflammatory response, and TGF-β1 treatments in tenocytes were adopted to analyze the fibrotic pathological process. EVs labeled with DII iodide were used in the EV uptake assay. The cell death status was analyzed with a Cytotoxicity Assay Kit (Beyotime, Hangzhou, China), and propidium iodide (PI) was used to stain dead cells with red fluorescence. The relative ratio of dead cells was used in the data analyses. The cellular senescence status was detected by two independent methods, including biological markers, including p21^Cip1^ and p16^INK4a^, and SA-β-gal staining. The cellular migration assay was conducted with a transwell test, and the migrated cells were stained with crystal violet. For the pathological fibrosis assay, α-SMA and phalloidin immunofluorescent staining of tenocytes in different groups was detected. Total collagen contents in the supernatants were analyzed using a mouse total collagen assay kit (DASF, Nanjing, China). The relative ratios of collagen contents compared with the control group were recorded and analyzed. The mRNA expression levels of fibrotic genes (COL I, COL III, Den and Cx43) and extracellular matrix remodeling-related genes (MMP1, MMP3, MMP9, TIMP1 and TIMP2) were evaluated using RT‒PCR. Mitochondrial membrane potential detection was conducted using a JC-1 kit (Cat. No. C2003S, Beytime, Hangzhou, China). The formation of aggregates by JC-1 produced red fluorescence, which indicated that the membrane potential was high, while JC-1 monomers indicated that the mitochondrial membrane potential was low and thus produced green fluorescence. Both the extracellular acidification rate (ECAR) and oxygen consumption rate (OCR) were used in cellular energy metabolism analyses. Tenocytes in different groups were seeded on Seahorse XF24 plates (Agilent Technologies, Santa Clara, CA, USA) at 1 × 10^4^ cells/well. For OCR detection, 1 μM oligomycin, 2 μM FCCP, and 2 μM Rot/AA were added to the activated probe. Another plate of cells was placed into the prewarmed Seahorse XFe24 Analyzer (Agilent Technologies, Santa Clara, CA, USA) for ECAR detection. The detection solution was heated to 37 °C, the pH was adjusted to 7.4, the cells were washed twice with 1 mL of detection solution, and then 10 mM glucose, 1 μM oligomycin, and 50 mM 2-DG were added to the activated probe. The results were calculated and analyzed according to the obtained real-time detection values of ECAR and OCR. ATP production and LDH release in cultured cells were detected using an ATP Assay Kit (Beyotime, Hangzhou, China) and LDH Release Assay Kit (Beyotime, Hangzhou, China), respectively. A phagocytosis assay was conducted to analyze the phagocytic functions in macrophages with different treatments.

Macrophage M2 polarization was identified using both flow cytometry with CD206 antibody (Thermo Fisher Scientific, USA) and the expression of M1/M2 markers (CD86, iNOS, IL-6, TLR4, Arg-1, Fizz-1, Ym-1 and CD206). The senescence-associated secretory phenotype (SASP) was identified by detecting the mRNA expression of related key factors including IL-6, IL-8, TGF-β1, MCP-1, MMP3, TNF-α and CXCL1. The Nile red fluorescent beads at a concentration of 5 × 10^5^ beads/ml (Invitrogen, USA) were incubated with macrophages for 2 h. After two washes with PBS, the extracellular beads were removed. Then, the cellular samples were fixed with 4% PFA for 15 min, stained with DAPI, and observed under a fluorescence microscope.

### Signaling experiments

To detect the signaling pathways of the protective effects of EVs in tenocytes and macrophages, 100 ng/mL recombinant IL-1β (Sino Biological Inc., Beijing, China) was used in this study. The potential roles of the NAMPT/SIRT1/PPARγ/PGC-1α pathway in tenocytes and the NAMPT/SIRT1/Nf-κb p65/NLRP3 pathway in macrophages were analyzed. NAMPT inhibitor FK566 (10 nM) or SIRT1 inhibitor EX-527 (100 nM) pretreatments were conducted to study the roles of NAMPT and SIRt in the signaling pathway.

### Immunofluorescence.

Tenocytes and macrophages in logarithmic phase were digested into single-cell suspensions, and the plated cells were used in advanced experiments when grown to 70–80% confluence. The cells in different groups were treated for 24 or 48 h and then used in immunofluorescence assays. After washing with PBS and fixing with 4% paraformaldehyde, the p16^INK4A^ (1:200, ab211542, Abcam, UK), p21^CIP1^ (1:200, 10355-1-AP, Proteintech, USA) and α-SMA (1:200, 19245, CST, USA) primary antibodies were added and incubated overnight at 4 ℃. After incubating with the fluore scently labeled secondary antibody (Thermo Fisher Scientific, USA) at room temperature for 2 h, the cell plates were washed with PBS. DAPI incubation was conducted for nuclear staining at room temperature for 2 min, and finally, the cells were observed with a fluorescence microscope (IX53, Olympus, Tokyo, Japan). Cytoskeleton staining was conducted with a phalloidin product (A12379, Thermo Fisher Scientific, USA) according to the manufacturer’s instructions.

### RT‒PCR

RT‒PCR was adopted to detect the expression of key genes involved in NAD+ metabolism, macrophage polarization, SASP status and extracellular matrix remodeling. Total RNA samples from cultured ADMSCs, tenocytes and macrophages in each group were extracted with TRIzol reagent (Invitrogen, USA). After removing the mixed DNA and DNase, quantification of extracted RNA was conducted by spectrophotometry (Nanodrop 2000, Invitrogen, USA). First-strand cDNA was synthesized from total RNA with a Fast King RTPCR synthesis kit (Tiangen, China). RT‒PCR was performed with the FastQuant RT Kit (Tiangen, China), and the reaction program was as follows: predenaturation at 95 °C for 15 min; denaturation at 95 °C for 10 s, annealing at 55 °C for 20 s, and extension at 72 °C for 30 s, 40 cycles. β-actin was adopted as an internal reference. The relative expression of mRNA was calculated by the 2^−ΔΔCt^ method. The primers are listed in Additional file 1: Table S1.

### Western blot

ADMSCs, tenocytes and macrophages in different groups were used in protein content assays. RIPA lysis buffer was used for total protein extraction, and the protein concentration was determined by a BCA kit (Beyotime. Hangzhou, China). After adding loading buffer, the extracted protein samples were boiled and used for advanced experiments. Then, the proteins were separated by SDS‒PAGE and primary antibodies, including NMAPT (1:1000, ab236874, Abcam, UK), SIRT1 (1:500, 2028S, CST, USA), PPARγ (1:1000, ab272718, Abcam, UK), PGC-1α (1:500, ab106814, Abcam, UK), p65 K310Ac (1:1000, ab19870, Abcam, UK), NLRP3 (1:1000, 19771-1-AP, Proteintech, USA), ASC (1:1000, 10,500-1-AP, Proteintech, USA) and β-actin (1:1000, Cat. No. 3700, CST, USA) at 4 ℃ overnight. The membranes were incubated with secondary antibody for 2 h at room temperature. ECL chemiluminescence was used to detect the signal. β-actin was adopted as an internal reference, and the bands were quantitatively analyzed by ImageJ software.

### Statistical analysis

The experimental data were analyzed using GraphPad 8.4.3 software (GraphPad Software Inc., CA, USA). Continuous data are expressed as the mean ± standard deviation. Student’s t test was used to analyze the difference between two groups, and one-way analysis of variance (ANOVA) was used to compare the means among multiple groups. The SNK test was used to compare the means of each group pairwise if there were significant differences. A P < 0.05 was considered statistically significant.

## Supplementary Information

**Additional file 1: Figure S1.** Identification of ADMSCyoung and ADMSCold by flow cytometric analysis by surface markers of ADMSCs.**Figure S2.** H&E staining of live and kidney cells in the control, TGF-β1, TGF-β1+ADMSCyoung-EV and TGF-β1+ADMSCold-EV tenocyte groups 4 weeks after treatment. Scale bar = 100 μm. **Figure S3.** Blood biochemistry examination of the control, TGF-β1, TGF-β1+ADMSCyoung-EV and TGF-β1+ADMSCold-EV tenocyte groups 4 weeks after treatment, n = 4. Blood chemistry data suggested no hepatic disorder induced by any EV treatment. Data are presented as the mean ± SD (ns: no significance). **Figure S4.** The expressions of SIRT1 in ADMSCyoung and ADMSCold were measured by western blotting.**Table S1.** The primers for RT-PCR assay in this experiment.

## Data Availability

All data needed to evaluate the conclusions in the paper are present in the paper and/or the Supplementary Materials.
