# Supplementary material for: NAMPT encapsulated by extracellular vesicles from young adipose-derived mesenchymal stem cells treated tendinopathy in a “One-Stone-Two-Birds” manner
Source: J Nanobiotechnology. 2023 Jan 5;21:7. doi: 10.1186/s12951-022-01763-5 (PMC9814467; doi:10.1186/s12951-022-01763-5)
Supplement: Supplementary file 1 — Additional file 1: Figure S1. Identification of ADMSCyoung and ADMSCold by flow cytometric analysis by surface markers of ADMSCs.Figure S2. H&E staining of live and kidney cells in the control, TGF-β1, TGF-β1+ADMSCyoung-EV and TGF-β1+ADMSCold-EV tenocyte groups 4 weeks after treatment. Scale bar = 100 μm. Figure S3. Blood biochemistry examination of the control, TGF-β1, TGF-β1+ADMSCyoung-EV and TGF-β1+ADMSCold-EV tenocyte groups 4 weeks after treatment, n = 4. Blood chemistry data suggested no hepatic disorder induced by any EV treatment. Data are presented as the mean ± SD (ns: no significance). Figure S4. The expressions of SIRT1 in ADMSCyoung and ADMSCold were measured by western blotting.Table S1. The primers for RT-PCR assay in this experiment. [file 12951_2022_1763_MOESM1_ESM.docx]

Supplementary Materials:


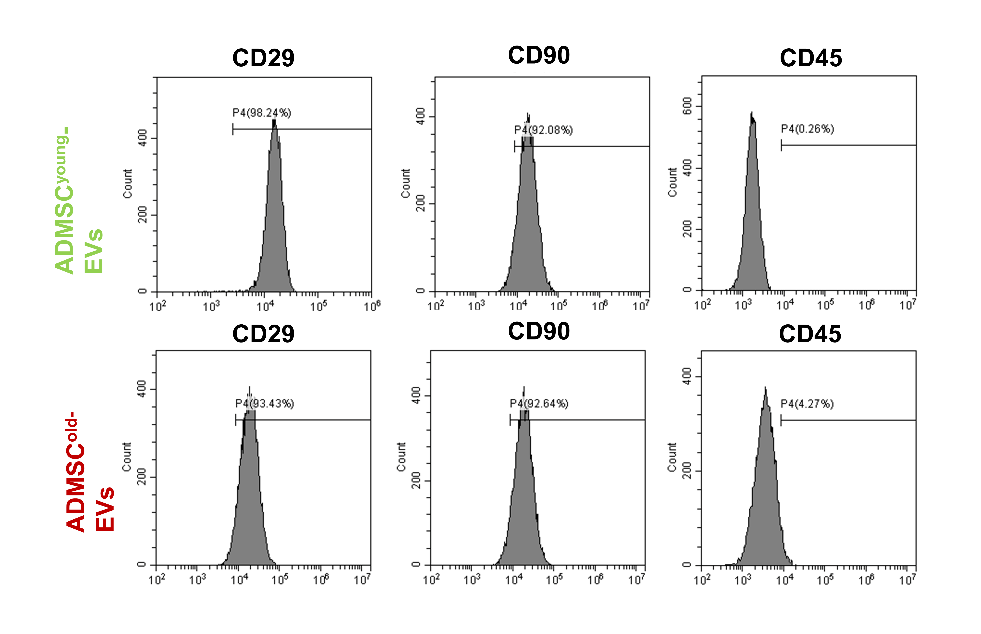


**Fig. s1**. Identification of ADMSC^young^ and ADMSC^old^ by flow cytometric analysis by surface markers of ADMSCs.


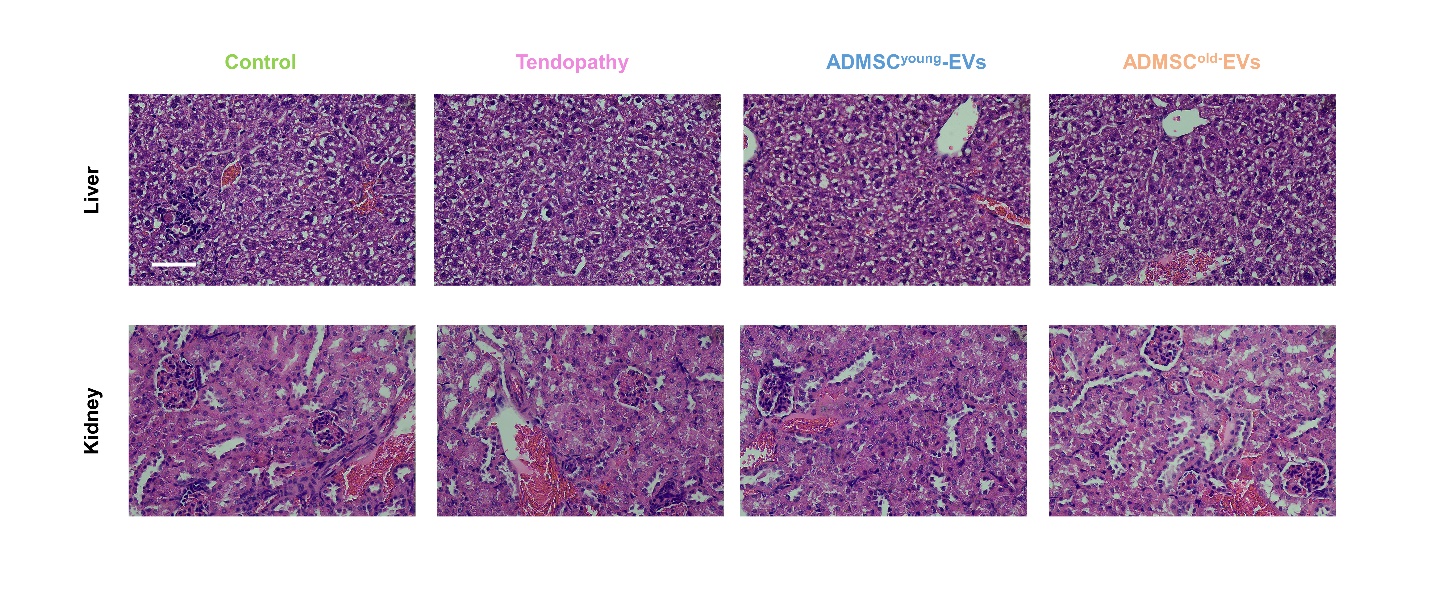


**Fig. s2**. H&E staining of live and kidney cells in the control, TGF-β1, TGF-β1+ADMSC^young^-EV and TGF-β1+ADMSC^old^-EV tenocyte groups 4 weeks after treatment. Scale bar = 100 μm


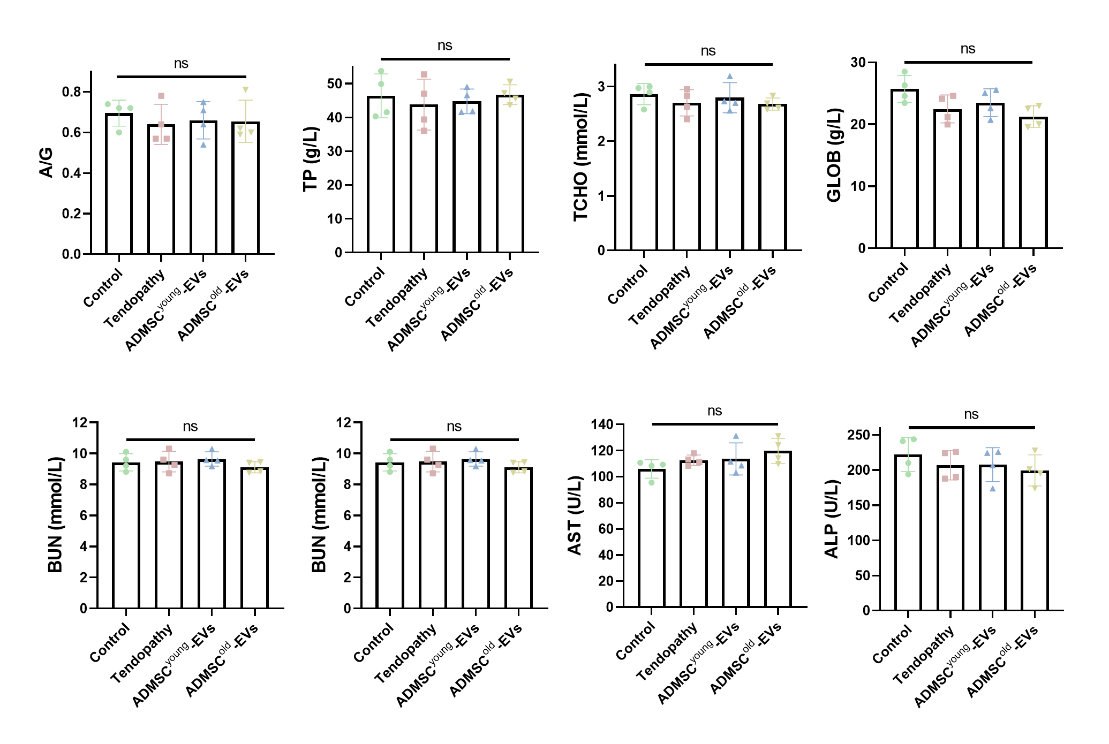


**Fig. s3**. Blood biochemistry examination of the control, TGF-β1, TGF-β1+ADMSC^young^-EV and TGF-β1+ADMSC^old^-EV tenocyte groups 4 weeks after treatment, n = 4. Blood chemistry data suggested no hepatic disorder induced by any EV treatment. Data are presented as the mean ± SD (ns: no significance).


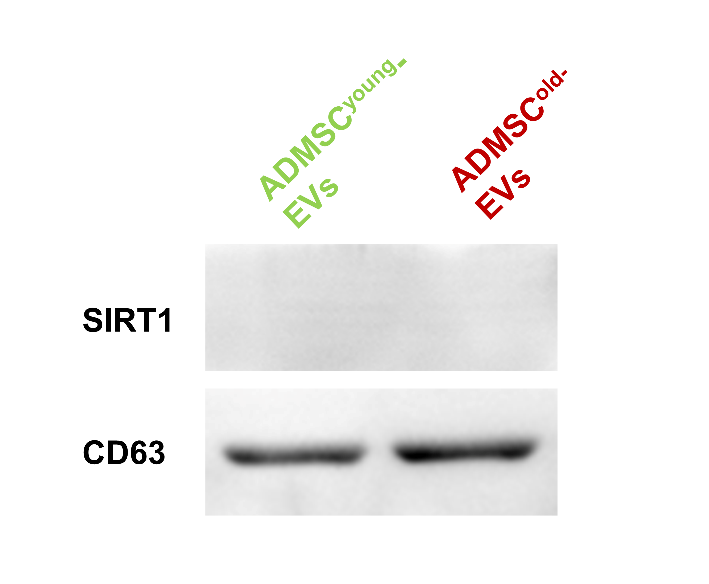


**Fig. s4**. The expressions of SIRT1 in ADMSC^young^ and ADMSC^old^ were measured by western blotting.

**Supplemental Table 1. The primers for RT-PCR assay in this experiment.**

| Gene | Oligonucleotide sequence (5’-3’) |
| --- | --- |
| COX2 | Forward, ATAACCGAGTCGTTCTGCCAAT  Reverse, TTTCAGAGCATTGGCCATAGAA |
| 18s rRNA | Forward, AGGGGAGAGCGGGTAAGAGA  Reverse, GGACAGGACTAGGCGGAACA |
| TNF-α | Forward, TTCTCATTCCTGCTTGTGG  Reverse, CTTGGTGGTTTGCTACGAC |
| IL1β | Forward, CAGTGGCAATGAGGATGACTTG  Reverse, GTAGTGGTGGTCGGAGATTCGTA |
| IL18 | Forward, ACGCTTTACTTTATAGCTGAAGATG  Reverse, GAGGCCGATTTCCTTGGTCA |
| MCP-1 | Forward, AGCCACCTTCATTCCCCAAG  Reverse, TTGGGTTTGCTTGTCCAGGT |
| CXCL1 | Forward, CACTGTGATAGAGGCTGGCG  Reverse, TCCAGTAAAGGTAGCCCTTGT |
| CXCL2 | Forward, AGATCAATGTGACGGCAGGG  Reverse, TCTCTGCTCTAACACAGAGGGA |
| β-actin | Forward, ACAGAGCCTCGCCTTTGCC  Reverse, TGGGGTACTTCAGGGTGAGG |
| CD86 | Forward, TCTGCCGTGCCCATTTACAAAGG  Reverse, TGTGCCCAAATAGTGCTCGTACAG |
| iNOS | Forward, TAGGCAGAGATTGGAGGCCTTG  Reverse, GGGTTGTTGCTGAACTTCCAGTC |
| IL-6 | Forward, CAACGATGATGCACTTGCAGA  Reverse, CTCCAGGTAGCTATGGTACTCCAGA |
| TLR4 | Forward, CGCTCTGGCATCATCTTCAT  Reverse, GTTGCCGTTTCTTGTTCTTCC |
| Arg-1 | Forward, CCCCAGTACCAACAGGACTACC  Reverse, TGAACGTGGCGGAATTTTGT |
| Fizz-1 | Forward, GGAACTTCTTGCCAATCCAGC  Reverse, AAGCCACAAGCACACCCAGT |
| Ym-1 | Forward, GAAGGAGCCACTGAGGTCTG  Reverse, GAGCCACTGAGCCTTCAAC |
| CD206 | Forward, GGGACTCTGGATTGGACTCA  Reverse, CCAGGCTCTGA TGATGGACT |
| MMP1 | Forward, TGCCTGATGTGGGTGAATAC  Reverse, GCCTTTGGAACTGCTTGTC |
| MMP9 | Forward, AGACCAAGGGTACAGCCTGTTC  Reverse, GGCACGCTGGAATGATCTAAG |
| TIMP1 | Forward, GCCTACACCCCAGTCATGGA  Reverse, GGCCCGTGATGAGAAACTCTT |
| TIMP2 | Forward, GTCCCATGATCCCTTGCTACA  Reverse, TGCCCATTGATGCTCTTCTCT |
| COL I | Forward, CCACCCCAGCCGCAAAGAGTC  Reverse, GTCATCGCACACAGCCGTGC |
| COL III | Forward, CCTGGAGCCCCTGGACTAATAG  Reverse, GCCCATTGCACCAGGTTCT |
| Dcn | Forward, FCAACAACAAACTCCTCAGGGTGC  Reverse, RTTGCCGTAAAGACTCACAGCCG |
| Cx43 | Forward, TTGGTGTCTCTCGCTCTGAAT  Reverse, CGCTGATCCACGATAGCTAAG |
| IL-8 | Forward, CAGCTGCCTTAACCCCATCA  Reverse, CTTGAGAAGTCCATGGCGAAA |
| TGF-β1 | Forward, TGGAGCAACATGTGGAACTC  Reverse, GTCAGCAGCCGGTTACCA |
| MCP-1 | Forward, CGCCTCCAGCATGAAAGTCT  Reverse, GGGAATGAAGGTGGCTGCTA |
| MMP3 | Forward, CATGGAGACTTTGTCCCTTTTGAT  Reverse, CGTCAAAGTGAGCATCTCCATTA |
| TNF-α | Forward, ATCCGCGACGTGGAACTG  Reverse, ACCGCCTGGAGTTCTGGAA |
| CXCL1 | Forward, CACTGTGATAGAGGCTGGCG  Reverse, TCCAGTAAAGGTAGCCCTTGT |
| PARP7 | Forward, GCCAGACTGTGTAGTACAGCC  Reverse, GGGTTCCAGTTCCCAATCTTTT |
| NAMPT | Forward, GCGAGCGAGCGGTGACT  Reverse, CTGCGAGCAAGGAGAAAAATG |
| PARP9 | Forward, AGGACGCCAAAGGGATCTG  Reverse, CCGGCTCCATAAACTGGGT |
| PARP2 | Forward, GCAACAGAAGACGACTCTCCT  Reverse, CAGCCATAGGCCCTTTTCTCT |
| SIRT4 | Forward, GTCCCGTGCTGTGATCGA  Reverse, CGGGCGGTGAGGATGAAC |
| PARP3 | Forward, ATGGCTCCAAAACGAAAGGC  Reverse, TCCTCCTCTGTCCCTTGTCG |
| PARP6 | Forward, CCAGACCCGATCCATTCAGTC  Reverse, CACAGGGCACACCCAAATGT |
| SIRT1 | Forward, TGTTTCCTGTGGGATACCTGA  Reverse, TGAAGAATGGTCTTGGGTCTTT |
| PARP8 | Forward, TAAATCGCACAAACTTTTGGGC  Reverse, TCTCCAGAACAAGATCGAGTCAA |
| PARP1 | Forward, GGCAGCCTGATGTTGAGGT  Reverse, GCGTACTCCGCTAAAAAGTCAC |
| PARP12 | Forward, TCATCTACGGCAACTGCAAGT  Reverse, AGCTCAGTATATGTGAGGTGGTC |
| NNMT | Forward, TTACAGCTTTGGGTCCAGACA  Reverse, GGAGTTCTCCCTTTACAGCAC |
| PARP11 | Forward, GTGGACGACATGGACACATCG  Reverse, CCAGTGACAAGATTCATCTGCTT |
| PARP14 | Forward, TGCCAAGCAGTCAGTGATGTC  Reverse, CCTGGAAAACTGTGTGCTCTAT |
| SIRT6 | Forward, ACGCGGATAAGGGCAAGT  Reverse, CTCCCACACCTTGCGTTC |
| KMO | Forward, ATGGCATCGTCTGATACTCAGG  Reverse, CCCTAGCTTCGTACACATCAACT |
| TNKS | Forward, GTCTACTCCGTTACACCTGGC  Reverse, TGAAGAGGTACAAGTCCACCTTT |
| SIRT3 | Forward, GCTGCTTCTGCGGCTCTATAC  Reverse, TGCTCCCCAAAGAACACAATG |
| TDO2 | Forward, ATGAGTGGGTGCCCGTTTG  Reverse, GGCTCTGTTTACACCAGTTTGAG |
| PARP10 | Forward, CTCTGTACTTTGAAAACCACCGT  Reverse, GCTCAGTCGGACACCATGTA |
| SIRT5 | Forward, GCCTCCCCACAAAGCAAGA  Reverse, AACCCCACTCTCCGCACTAA |
| NMNAT3 | Forward, CCTGTGGTTCCTTCAACCCC  Reverse, AGATGATGCCCTCAATCACCT |
| CD157 | Forward, AGGGACAAGTCACTGTTCTGG  Reverse, AACTTTGCCATACAGCACGTC |
| KYNU | Forward, GTCAAGCCTGCGTTAGTGG  Reverse, GGAGGGTTTGAAATTCGGAATCC |
| SIRT2 | Forward, CAAGCCAACCATCTGCCACTA  Reverse, CCCGCCACTCGTTCCA |
| IDO2 | Forward, TGGGGAGATACCACATTTCTGA  Reverse, TGAGGAAGTCTGAGGGCAATTT |
| IDO1 | Forward, GCTTTGCTCTACCACATCCAC  Reverse, CAGGCGCTGTAACCTGTGT |
| PARP4 | Forward, TCATACCACCTAAGTTGGGTCC  Reverse, AGCAAAGCTACCTGAGAAAGC |
| NMNAT1 | Forward, TGGCTCTTTTAACCCCATCAC  Reverse, TCTTCTTGTACGCATCACCGA |
| NAPRT | Forward, TGCTCACCGACCTCTATCAGG  Reverse, CGAAGGAGCCTCCGAAAGG |
| NMNAT2 | Forward, ATGACCGAGACCACAAAGACC  Reverse, ATCCCGCCAATCACAATAAATCT |
| CD38 | Forward, TCTCTAGGAAAGCCCAGATCG  Reverse, GTCCACACCAGGAGTGAGC |
| SIRT7 | Forward, GCCAGGAGGAGGTGTGTGA  Reverse, GGCTCCGCTTCGCTTAGGT |
| HAAO | Forward, GAACGCCGTGTGAGAGTGAA  Reverse, CCAACGAACATGATTTTGAGCTG |

COX2: Cytochrome oxidase subunit 2; TNF-α: tumor necrosis factor-α; IL1β: interleukin 1β; IL8: interleukin 8; IL18: interleukin 18; MCP-1: monocyte chemoattractant protein 1; CXCL1: chemokine ligand 1; CXCL2: chemokine ligand 2;

iNOS: nitric oxide synthase 2, inducible; IL-6: interleukin 6; TLR4: toll like receptor 4; Arg-1: arginase-1; Fizz-1: resistin-like molecule alpha 1; Ym-1: chitinase-like 3; MMP1: matrix metallopeptidase 1; MMP9: matrix metallopeptidase 9; TIMP1: tissue inhibitor of metalloproteinase 1; TIMP2: tissue inhibitor of metalloproteinase 2; COL I: collagen type I; COL III: collagen type III; Dcn: decorin; Cx43: connexin 43; IL-8: interleukin 8; TGF-β1: transforming growth factor beta 1; MCP-1: chemokine (C-C motif) ligand 2; MMP3: matrix metallopeptidase 3; TNF-α: tumour necrosis factor alpha; CXCL1: C-X-C motif chemokine ligand 1; PARP7: TCDD inducible poly(ADP-ribose) polymerase; NAMPT: nicotinamide phosphoribosyltransferase; PARP9: poly(ADP-ribose) polymerase family member 9; PARP2: poly(ADP-ribose) polymerase 2; SIRT4: sirtuin 4; PARP3: poly(ADP-ribose) polymerase family member 3; PARP6: poly(ADP-ribose) polymerase family member 6; SIRT1: sirtuin1; PARP8: poly(ADP-ribose) polymerase family member 8; PARP1: poly(ADP-ribose) polymerase 1; PARP12: poly(ADP-ribose) polymerase family member 12; NNMT: nicotinamide N-methyltransferase; PARP11: poly(ADP-ribose) polymerase family member 11; PARP14: poly(ADP-ribose) polymerase family member 14; SIRT6: sirtuin6; KMO: kynurenine 3-monooxygenase; TNKS: tankyrase; SIRT3: sirtuin3; TDO2: tryptophan 2,3-dioxygenase; PARP10: poly(ADP-ribose) polymerase family member 10; SIRT5: sirtuin5; NMNAT3: nicotinamide nucleotide adenylyltransferase 3; KYNU: kynureninase; SIRT2: sirtuin2; IDO2: indoleamine 2,3-dioxygenase 2; IDO1: indoleamine 2,3-dioxygenase 1; PARP4: poly(ADP-ribose) polymerase family member 4; NMNAT1: nicotinamide nucleotide adenylyltransferase 1; NAPRT: nicotinate phosphoribosyltransferase; NMNAT2: nicotinamide nucleotide adenylyltransferase 2; SIRT7: sirtuin7; HAAO: 3-hydroxyanthranilate 3,4-dioxygenase.
